# Proteins mediating DNA loops effectively block transcription

**DOI:** 10.1002/pro.3156

**Published:** 2017-03-27

**Authors:** Zsuzsanna Vörös, Yan Yan, Daniel T. Kovari, Laura Finzi, David Dunlap

**Affiliations:** ^1^ Department of PhysicsEmory UniversityAtlantaGeorgia30322

**Keywords:** transcription elongation, RNAP, lac repressor protein, DNA looping, scanning force microscopy, magnetic tweezers

## Abstract

Loops are ubiquitous topological elements formed when proteins simultaneously bind to two noncontiguous DNA sites. While a loop‐mediating protein may regulate initiation at a promoter, the presence of the protein at the other site may be an obstacle for RNA polymerases (RNAP) transcribing a different gene. To test whether a DNA loop alters the extent to which a protein blocks transcription, the lac repressor (LacI) was used. The outcome of in vitro transcription along templates containing two LacI operators separated by 400 bp in the presence of LacI concentrations that produced both looped and unlooped molecules was visualized with scanning force microscopy (SFM). An analysis of transcription elongation complexes, moving for 60 s at an average of 10 nt/s on unlooped DNA templates, revealed that they more often surpassed LacI bound to the lower affinity O2 operator than to the highest affinity Os operator. However, this difference was abrogated in looped DNA molecules where LacI became a strong roadblock independently of the affinity of the operator. Recordings of transcription elongation complexes, using magnetic tweezers, confirmed that they halted for several minutes upon encountering a LacI bound to a single operator. The average pause lifetime is compatible with RNAP waiting for LacI dissociation, however, the LacI open conformation visualized in the SFM images also suggests that LacI could straddle RNAP to let it pass. Independently of the mechanism by which RNAP bypasses the LacI roadblock, the data indicate that an obstacle with looped topology more effectively interferes with transcription.

## Introduction

Proteins bound to DNA may act as roadblocks that hinder elongation by RNA polymerase during transcription. The mechanisms by which RNA polymerases might surpass such roadblocks are poorly understood. Previous single‐molecule studies have focused on RNA polymerases disrupting nucleosomes.^1^, ^2^, ^3^, ^4^ However, nucleosomes are only found in eukaryotes, interact with DNA nonspecifically, and are substrates for post‐translational modifications that regulate chromatin remodeling and the recruitment of accessory factors that regulate transcription.^5^ In contrast, many transcription factors from organisms spanning all kingdoms recognize specific sites on DNA to shape the genome and regulate various genomic functions, and may or may not undergo chemical modifications regulated by complex pathways. Very often they recognize multiple specific sequences to which they bind with different affinities and cooperatively.^6^, ^7^ These tunable, cooperative interactions determine ubiquitous architectural DNA modifications such as DNA looping, the role of which has not been directly investigated in earlier studies on transcription roadblocks either in vivo or in vitro.

While a loop‐mediating protein may regulate initiation from the promoter near one of the bridged binding sites, its presence at the other site may be an obstacle for RNA polymerases transcribing a different gene. To test whether a DNA loop alters the extent to which a protein halts transcription, the lac repressor protein (LacI) was used. LacI is a paradigmatic DNA looping transcription factor which in *E. coli*, may bind cooperatively to two of three operators inducing a DNA loop to efficiently repress the expression of three genes involved in the metabolism of lactose.^8^, ^9^ LacI can bind to a high affinity site adjacent to the promoter (O1), a ∼four‐fold lower affinity, secondary site ∼400 bp downstream (O2), and an ∼67‐fold lower affinity, tertiary site ∼90 bp upstream (O3).^10^, ^11^, ^12^ A strong, symmetric operator, Os, which LacI binds with five‐fold greater affinity than O1, has also been engineered.^13^ The auxiliary sites are thought to serve as reservoirs to elevate the concentration of LacI in proximity to the primary, promoter‐blocking site by forming loops to deliver LacI to the primary site.^14^ In addition, LacI tetramers exhibit higher affinity for operators in looped as compared to unlooped DNA.^15^ LacI bound to an operator can block transcription initiation with up to 99.5% efficiency in vivo^16^, ^17^ that depends on the promoter firing rate and whether multiple RNA polymerases act cooperatively.^18^ Lac repressor can also obstruct eukaryotic RNA polymerase II^19^ to a greater or lesser degree depending on accessory factors.^20^

The extent to which a DNA loop accentuates the repression of transcription initiation at the lac operon has been amply characterized; however, its strength as a roadblock to an advancing transcription elongation complex has never been characterized. Therefore, in this study, the progress of RNA polymerase through LacI obstacles on torsionally relaxed, looped or unlooped DNA templates was monitored using scanning force microscopy (SFM) and magnetic tweezers (MT). As expected based on reports in the literature,^14^ RNA polymerase more easily surpassed LacI obstacles on lower versus higher affinity operators in unlooped DNA molecule. Surprisingly, this difference was abrogated in looped DNA molecules where LacI became a strong roadblock independently of the affinity of the operator. This is remarkable in that a LacI bound to a lower affinity, O2 operator in a looped DNA molecule blocks RNA polymerase as effectively as one bound to an approximately 20‐fold higher affinity, Os operator. Furthermore, the open and closed LacI conformations were equally good roadblocks in looped DNA templates. The data indicate that an obstacle with looped topology more effectively interferes with transcription.

## Results

### Measuring the probability of transcriptional progress using scanning force microscopy

#### Sample preparation

**Figure 1:**
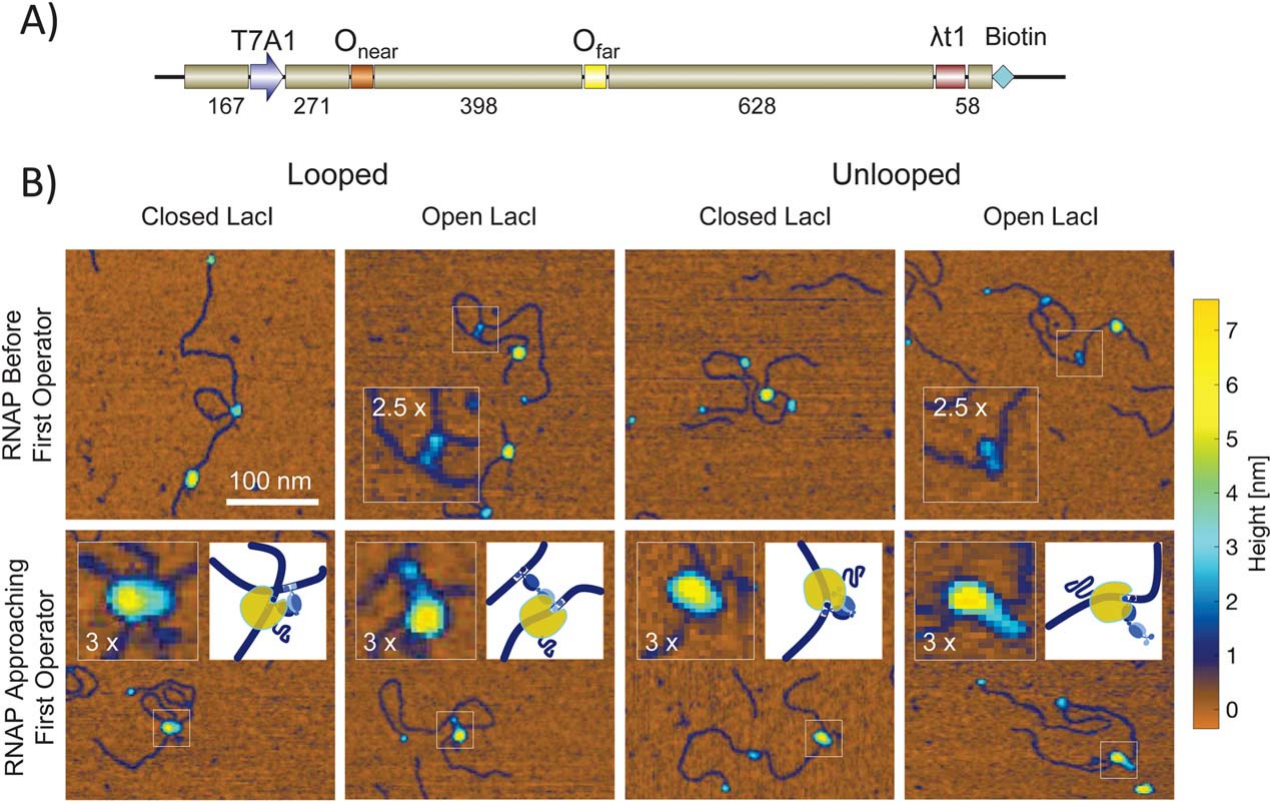
Nanographs of RNA polymerases trapped by EDTA quenching during elongation along DNA with and without LacI‐mediated loops. (A) Schematic representation of the DNA templates used in scanning force microscopy assays. All templates contained a T7A1 promoter close to the upstream end, a stall site at position +22, a “far” O1 operator, the lambda t1 terminator (λt1) and a biotin label at the downstream end. The two DNA templates used for SFM measurements of transcription differed in the “near” operator positioned 253 bp downstream from the promoter; one template contained the Os operator while the other contained the O2 operator. The terminator was the very last feature of the sequence and was biotin labeled. Streptavidin was coupled to the biotin label to facilitate identifying the “downstream” end of the molecule in SFM nanographs. (B) The upper row is a selection of molecules along which RNA polymerases (large yellow particle) had not progressed very far from the transcription start site near the end of the DNA without a streptavidin particle (blue). Closed and open conformations of the LacI tetramers are visible for either looped (left) or unlooped (right) columns. The closed conformations are shown as blue particles that are slightly larger than the streptavidin. In the open conformation, two lobes are visible especially on looped DNA. These lobes correspond to individual dimers with DNA binding head groups. The TECs shown in the lower row had progressed further and small coils of RNA emanate from them (see inset schematics for the regions of interest). These TECs have collided with LacI particles. The LacI particles correspond to blue protuberances on the periphery of the larger yellow RNA polymerase particle. The RNA polymerases themselves appear to shift to the side opposite LacI especially for open LacI conformations.

Scanning force microscopy was used to accurately correlate the disposition of RNA polymerase and LacI obstacles with the looped or unlooped configuration of the DNA molecule. Bound LacI is a roadblock for transcription, and reports have shown that its strength depends on the binding affinity for the operator.^16^, ^17^, ^18^ DNA molecules contained two operators for LacI separated by 400 bp, so that a *lac* repressor could mediate looping with negligible strain in the intervening DNA segment [Fig. 1(A)]. These molecules included either the high affinity Os or the 20‐fold lower affinity O2 operator 271 bp from the transcription start site (TSS). In both constructs an O1 operator was included 669 bp from the TSS. Transcription was activated using either template with or without LacI by adding 100 µM NTPs and incubating for 60 s. EDTA was added to interrupt transcription without dissociating RNA polymerase from the template.^21^ The sample was immediately deposited on poly‐L‐ornithine‐coated mica, incubated 1–2 min, rinsed, and dried for imaging.

#### LacI has two conformations

Slow scans (5 µm/s) over large (5 × 5 µm) areas produced images of hundreds of DNA molecules with and without bound RNA polymerase and/or lac repressor. Most had a bound streptavidin, which was used to mark the biotinylated downstream end of the DNA template. About 50% of the DNA molecules displayed a, large, bound RNA polymerase and a high percentage of these also displayed smaller LacI particles. Different topologies were observed with both parallel and anti‐parallel orientations of the DNA segments and examples of many of the conformations described previously (Supporting Information Fig. S1).^22^

The LacI particles could be catalogued as either in the closed or open conformations^22^, ^23^ that were especially clear in DNA molecules with LacI‐mediated loops [Fig. 1(B)]. Each of two lobes of the tetrameric lac repressor attaches to separate DNA segments to cross link them and secure a loop. Surprisingly, there is considerable conformational flexibility in a LacI‐mediated loop that allows as much as 14 nm separation between the bound DNA segments. This is likely to be important especially for LacI to mediate loops in short and therefore stiff DNA segments such as the 93 bp loop between O3 and O1 in *E. coli*. It could also be important in the mechanism by which a transcription elongation complex (TEC) bypasses a LacI obstacle.

#### Speed of elongation

**Figure 2:**
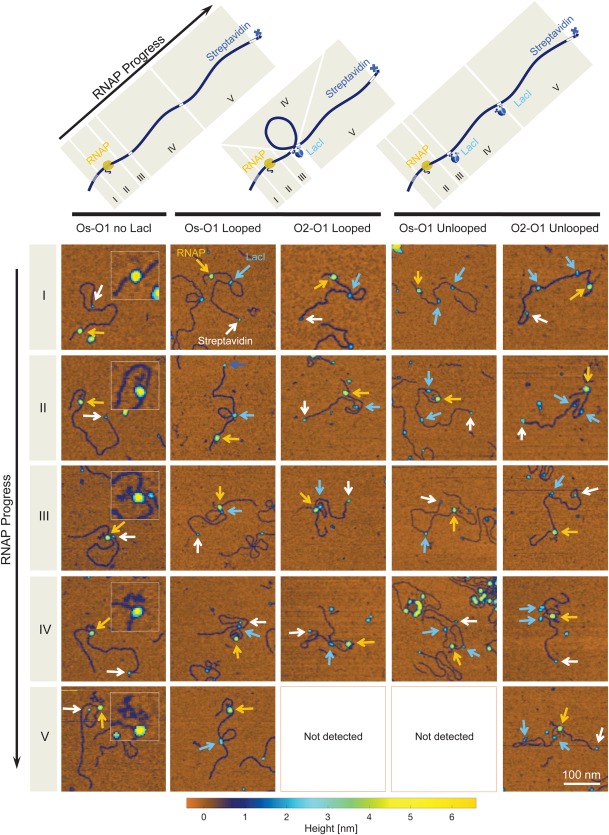
Nanographs of RNAP elongation along DNA with and without LacI‐mediated loops. (Top) Schematic representations of transcription elongation complexes (TECs). Columns correspond to different DNA topologies and LacI occupancy. The first column corresponds to transcription elongation without LacI in the reaction buffer. The second and third column correspond to DNA found in a looped topology, with LacI at each of the two operators; the fourth and fifth columns correspond to unlooped DNA with both operators occupied. Transcription elongation progress is categorized in five zones (roman numerals I‐V). Numerals in the schematic correspond to each row of the nanograph array. Each image in the array is representative of its corresponding category (columns) and elongation progress (rows). Image colors indicate height, according to the color scale below. RNAP, LacI and streptavidin particles are indicated by yellow, light blue, and white arrows respectively. (Row I) AFM images of RNAP bound at the T7A1 promoter. (Row II) Images in which TECs have not yet reached the near operator. (Row III) Images in which TECs contact LacI at the near operator. (Row IV) Images in which TECs were found between the two operators. (Row V) Images in which TECs were beyond the far operator. As indicated in the figure, images for RNAP in zone V were not detected for looped O2‐O1 DNA and unlooped Os‐O1 DNA. Note that nascent RNA associated with each TEC is visible, especially in the first column (insets), and increases in size as the RNAP progresses (I to V).

Aliquots were imaged to provide snapshots of the transcriptional progress made by RNA polymerase after 60 s of elongation with or without LacI obstacles. In the absence of LacI, transcription elongation complexes were found at various positions along the template (Fig. 2, left column; Supporting Information Fig. S2, top‐left). The schematic diagram above the column indicates five segments with which RNA polymerase positions were classified (see next section). The progress of the RNA polymerase is clearly due to transcription, because the nascent RNA grows as TECs are observed further along the template (Fig. 2 left column).

**Figure 3:**
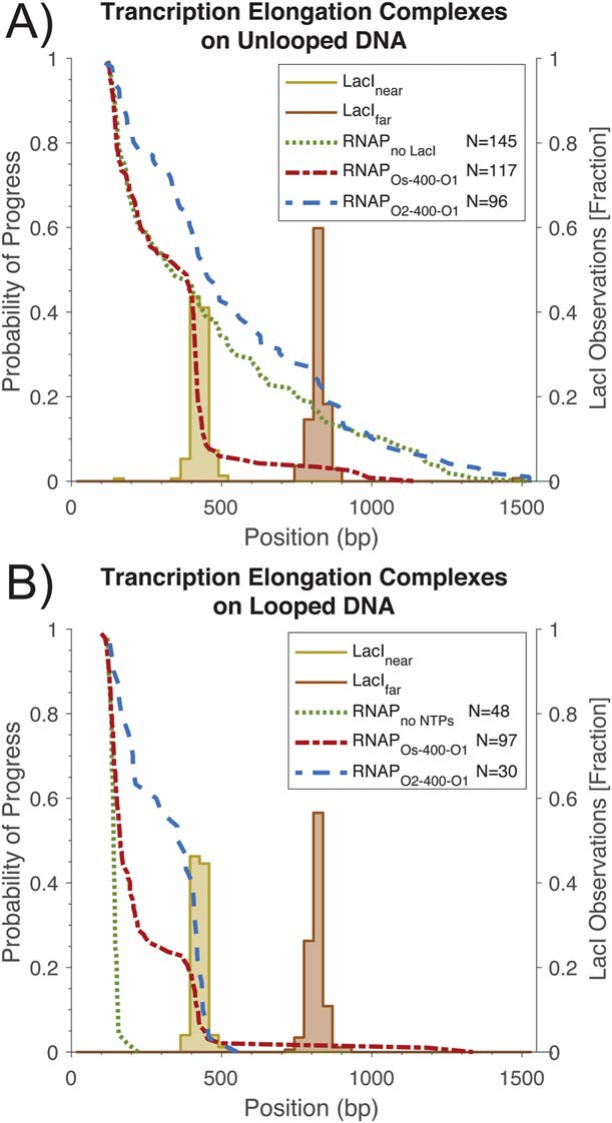
The probability of progress through obstacles. The positions of RNA polymerases and lac repressor molecules were measured along DNA contours in scanning force microscopy images. The probability of progress for each position was calculated as 1‐ [normalized cumulative histogram of RNAP position]. (See Supporting Information Fig. S2 for standard histograms of RNAP positions.) (A) Unlooped DNA templates. Without LacI obstacles (green dotted line), the majority of RNA polymerases began to transcribe (transcription elongation complexes, TECs) and after 60 s were distributed randomly along unlooped DNA templates. When LacI obstacles were present, a high percentage of TECs were blocked by LacI bound to the high affinity, Os operator (red, short‐dashed line). In contrast, a large percentage of TECs surpassed LacI bound to the lower affinity, O2 operator (blue, long‐dashed lines). (B) Looped DNA templates. Without NTPs, RNA polymerases were tightly bunched at the promoter site on DNA molecules with LacI‐mediated loops (green dotted line). Almost all TECs were blocked by LacI mediating loops between O1 and the high affinity, Os operator (red, short‐dashed line). Remarkably, LacI tetramers securing loops between O1 and the lower affinity, O2 operator, were equally effective obstacles (blue long‐dashed line). In each graph the yellow and orange histograms indicate the distributions measured for LacI along the DNA contours.

To calculate the average rate of transcription, a frequency distribution of the actual measured locations was plotted [Fig. 3(A), RNAP_no LacI_]. This plot shows the surviving fraction of TECs as a function of the distance along the template. Some TECs remain stuck at the promoter, but almost 50% transcribe far enough to reach the “near” operator which is unoccupied in this case. The surviving TECs progress along the DNA contour until they become randomly stopped or halted by the addition of EDTA (after 60 s). This distribution of observed positions was fitted with a Gaussian to describe the number of TECs stuck at promoters (inactive) and a decaying exponential function to describe the active, elongating population (Supporting Information Fig. S3). The average progress determined from this exponential function was 660 bp which, when divided by the incubation time, 60 s, produced an estimated average rate of elongation of 10.3 bp/s. This estimate agrees with previously reported in vitro rates at comparable NTP concentrations.^24^, ^25^, ^26^

#### LacI obstacles impede TECs

When LacI was added to the transcription assay, it formed roadblocks to transcription either bound to an operator along unlooped DNA, or bridging two operators to secure a loop. Additional series of nano‐topographs in Figure 2 show RNA polymerase at different stages of elongation along looped (columns 2 and 3) or unlooped (columns 4 and 5) templates. The rows of images are ordered according to the segments depicted in schematic diagrams above each column. RNA polymerases remaining at the T7A1 promoter are pictured in the first row (I), RNA polymerases that had not yet reached the near operator are in the second row (II), RNA polymerases in close proximity to the near operator follow in the next row (III), RNA polymerases between the two operators make up the fourth row (IV), and finally RNA polymerases that surpassed even the far operator are depicted in the bottom row (V).

The graphs in Figure 3 show the surviving fraction of TECs observed as a function of distance along the templates. These two graphs summarize the probability of progress observed for TECs encountering LacI obstacles on DNA templates with either Os or O2 in the “near” operator position, 271 bp from the promoter. Both templates have an O1 operator 669 bp from the promoter. In the absence of nucleotides, RNA polymerase was found only at the promoter in DNA complexes [Fig. 3(B), RNAP_no NTP_]. There are no surviving members of the ensemble beyond about 200 bp. The high specificity of promoter recognition displayed by RNA polymerase as well as the ability to follow elongation by monitoring the position along the DNA and the size of the nascent RNA, confirmed that elongation and roadblock effects could be studied by single molecule techniques.

Naïvely, one would expect that the ability of LacI to form a barrier to transcription depends on the affinity for the operator to which it is bound. Indeed, it is well known that LacI bound to the high affinity Os operator potently interferes with transcriptional elongation.^16^, ^17^, ^18^ TEC observations on unlooped DNA further illustrate this point. For that case, there is a significant difference in the fraction of TECs that surpass the low‐affinity O2 operator compared with the fraction that pass the high‐affinity Os operator [Fig. 3(A)]. LacI bound to an O2 operator only has a slight, if not negligible, effect on halting TEC progress; of the 96 measured TECs roughly 50% were found after the near (O2) operator (Supporting Information Fig. S2). In contrast, LacI bound to an Os operator blocks most of the TECs; only 10% surpass the obstacle.

#### Loops enhance interference with transcription

Contrary to the effect on LacI obstacles along unlooped DNA, operator affinity did not modulate obstruction in looped DNA. At 7.5 nM concentration, LacI begins to saturate the high affinity operators on a DNA template. Indeed LacI binding to operators was above 90% for the Os‐400‐O1 template and dropped about 20% with transcription. High occupancy of both operators blocks looping and reduces the percentage of looped molecules that can be achieved at lower concentrations.^27^ In SFM images, looped molecules constituted respectively 50 or 34% of the observations with or without transcriptional activity on the Os‐400‐O1 template (Supporting Information Figure S4). As expected, the fraction of LacI binding was lower for the O2‐400‐O1 template, as was the fraction of looped molecules, 14 or 12% with and without transcription. It is important to note for the analysis that follows, that the number of looped molecules did not decrease with transcription (+NTP).

Analysis of nano‐topographs like those in Figure 2 revealed that LacI‐mediated loops blocked elongation with almost 100% efficiency [Fig. 3(B)]. Whether the operator at the near position was the highest affinity Os or the 20‐fold lower affinity O2, LacI secured loops blocked TECs almost completely. Only a few of the TECs that reached the near LacI obstacle surpassed it even on an O2 operator. There was a difference in the occupancy of LacI at Os and O1 binding sites between the Os‐400‐O1 samples prepared with and without added NTPs. However, this did not change the ability of LacI bound at Os to effectively block RNA polymerase either in the looped or unlooped configuration. Furthermore, while the occupancy of O2 sites did not change when NTP was added, LacI bound at O2 was a much more effective barrier when it secured a loop.

This finding suggests that when the LacI tetramer is bivalently engaged to two operators, forming a loop, the effective affinity to both operators increases. This conclusion is further supported by the observation that activating transcription (through the addition of NTP to the reaction buffer) only slightly decreased the observed fraction of looped molecules on the O2‐400‐O1 DNA template and increased the looped fraction observed for the Os‐400‐O1 template (Supporting Information Fig. S4). It is worth noting that increased obstruction may not be solely attributable to increased affinity. In addition, the intersecting or apposed segments of DNA associated with a loop might prevent RNA polymerase from reaching a LacI obstacle. However, it is likely to be a small effect, since RNA polymerase in direct contact with the loop‐LacI complex appears in nanographs such as that depicted in Row III, column 1 of Figure 2.

### RNA polymerase overcomes pausing in magnetic tweezer experiments

#### Analysis of transcription using magnetic tweezers

**Figure 4:**
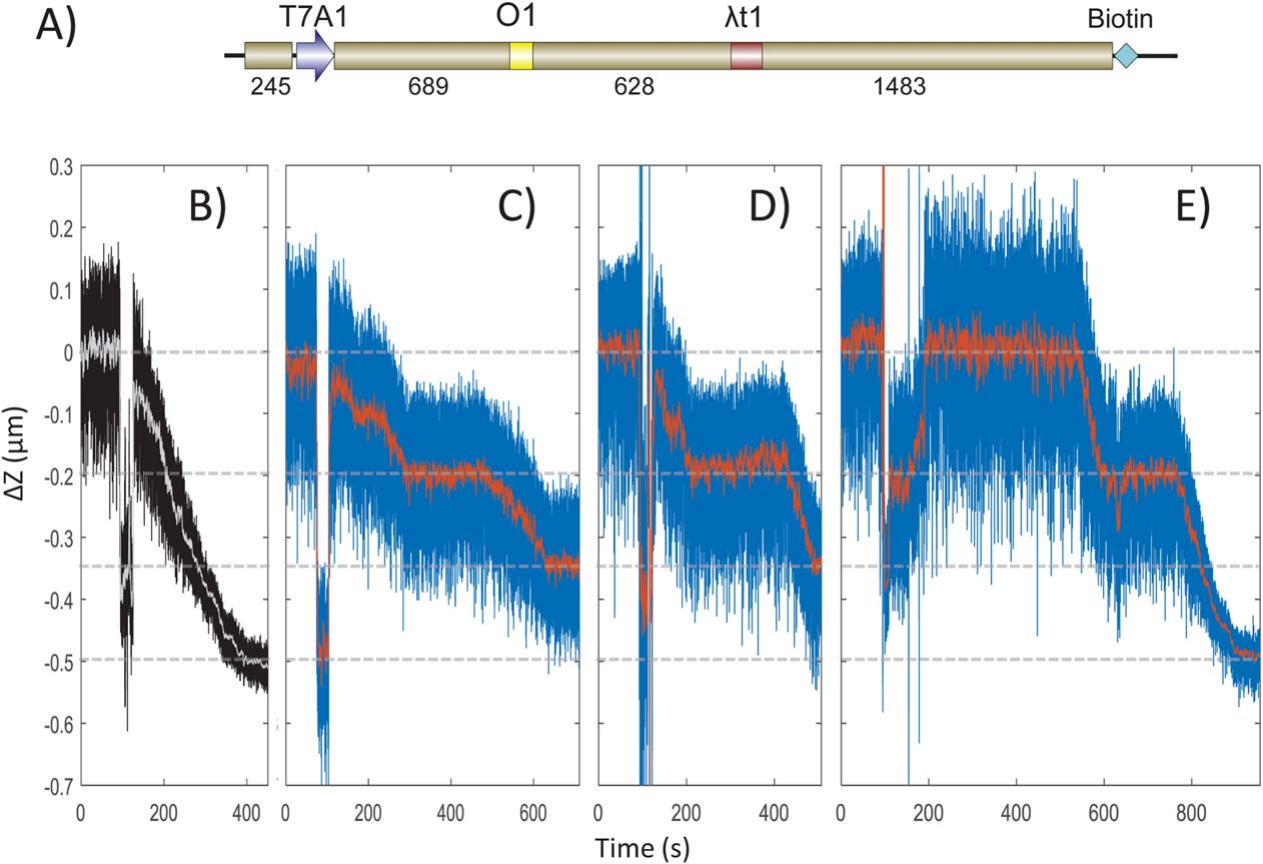
LacI bound to an O1 operator pauses transcription. (A) A schematic representation of the DNA template used in magnetic tweezer transcription assays. The template contained a T7A1 promoter close to the upstream end, a stall site at position +22, an O1 operator, the lambda t1 terminator (λt1) and a biotin label at the downstream end. A streptavidin‐labeled paramagnetic bead was coupled to the biotin label to for micromanipulation in the magnetic tweezer. Four examples of transcriptional elongation recorded using the magnetic tweezers are displayed. In **(**B**)** no LacI was included and transcription shortened the DNA tether progressively without interruption. When LacI was included **(**C–E**)**, transcription shortened the tether by about 0.2 um before pausing for about 200 s and then resuming. Transcription finally ceased after the tether shortened by either 0.35 um (C and D), a distance corresponding to the location of a terminator sequence, or 0.5 um (B and E), a distance corresponding to the end of the template.

To understand what happens to an RNA polymerase that encounters a LacI obstacle, the real‐time progress was tracked using magnetic tweezers (Supporting Information Fig. S5). A DNA tether containing only the O1 operator was used [Fig. 4(A)], and the change in the length of the tether was monitored after a missing nucleotide was added to allow stalled RNA polymerases to resume transcription. Figure 4(B–E) shows representative traces of the average change in the extension of DNA tethers as a function of time. The DNA extension is maximal at the beginning of the experiment when TECs were stalled at position +22 for lack of CTP [Fig. 4(B–E), top dashed line at 0]. When the missing nucleotide was added, elongation resumed after random delays and was detected as a progressive decrease in tether length after turbulence subsided.

**Figure 5:**
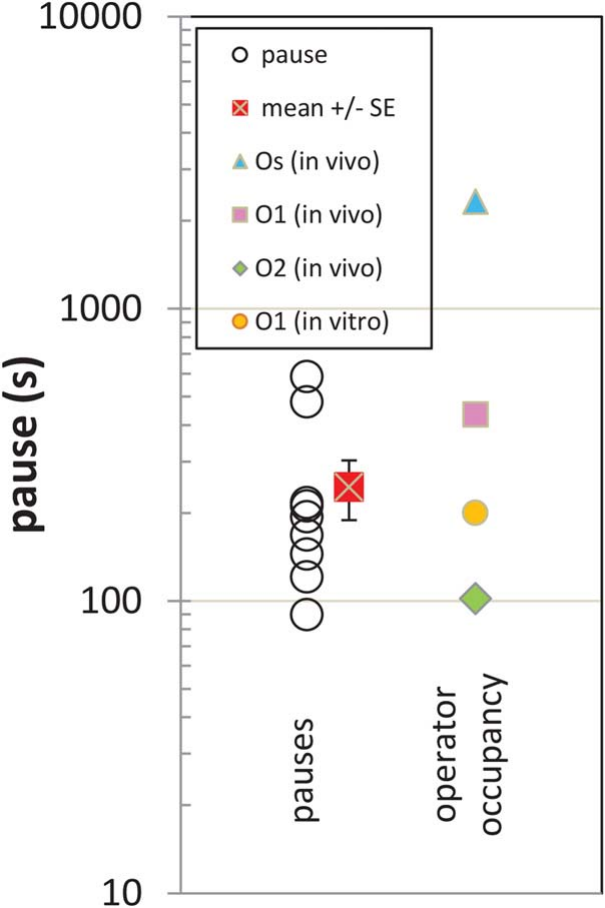
TEC pauses and expected operator occupancy. Sections of time series like those displayed in Figure 4 were fitted as described in Supporting Information Figure S6 to determine pause intervals at the position corresponding to the O1 operator site (○). The mean value is also plotted with standard error indicated (red x: 253). This mean is considerably shorter than expected the mean interval of occupancy of the operator in vivo calculated as the inverse of the reported dissociation rate of LacI from O1 (pink □: 434 s). Also shown are values for the expected mean intervals of *in vivo* occupancy of operators O2 (green ⋄: 102 s) and Os (blue Δ: 2326 s) and in vitro occupancy of O1 (orange circle: 200 s).

The leftmost record [Fig. 4(B) was in the absence of LacI and transcription proceeded smoothly consuming the entire tether, so that the bead was drawn down to the glass surface. Figure 4(C–E) show transcription progress in the presence of 1 or 10 nM LacI. Pauses in tether shortening (transcription elongation) were observed at the extension corresponding to O1 (dashed line at −0.2 µm). Pauses at the roadblock were measured and are reported in Figure 5 together with the mean lifetimes of LacI‐operator complexes estimated as the inverse of the measured off‐rates.^18^ Notice that the mean pause time is similar to the average dwell time of LacI on O1 in vitro. Furthermore, the average pause is longer than that measured previously for TECs with no obstacles, 90 ± 14 s.^28^ Sometimes transcription ceased at the terminator [Fig. 4(C,D), dashed line at −0.35 µm], but alternatively it continued until the bead was drawn down to the micro‐chamber surface [Fig. 4(B,E), dashed line at −0.5 µm].

## Discussion

### Operator occupancy is likely to be very high in SFM transcription assays

It is remarkable that RNA polymerase paused for one hundred or more seconds when it encountered LacI obstacles. Although this may not be relevant in vivo where additional factors like Mfd may relieve blockages and/or dissociate elongation complexes.^18^ it suggests that rather than dislodging obstacles, RNA polymerase simply waits for obstacles to disappear. This is compatible with the in vivo results in which promoter firing rate and the multiplicity of paused TECs affected read through.^18^ Such extended pauses indicate that during a transcription interval of 60 s, LacI can resist an advancing RNA polymerase particularly when bound to a high affinity operator like O1. For the large number of complexes analyzed using scanning force microscopy, LacI obstacles on Os operators are likely to endure throughout the interval. Given the lower affinity of LacI for O2, LacI obstacles on the unlooped O2‐400‐O1 templates may disappear intermittently to give RNA polymerase the opportunity to proceed, but this is probably negligible as described in the following paragraph and certainly occurs rarely in the looped conformation.

### Tethering and high LacI drive operator occupancy

Assaying the position of TECs using scanning force microscopy as described requires ruling out artifacts associated with the dynamic equilibrium of LacI binding. LacI might bind to an operator after a TEC has passed through, such that in a subsequent image the TEC will appear to have surpassed a LacI obstacle. Certainly after 30 min. of incubation (see Materials and Methods), LacI has established a dynamic equilibrium with operator sites along DNA molecules. However, 7.5 nM LacI fairly saturates Os operators (*K_d_* < 1 × 10^−14^ M in similar K^+^ conditions^29^), and the estimated tether‐augmented, effective concentration (*J* value) of LacI is expected to be in the range of 4 to 100 nM for a 400 bp loop (data not shown^30^). Therefore, operator occupancy is expected to be very high throughout the 60 s incubation. In addition, the large association rate constant determined for similar, in vitro conditions, 7.9 × 10^9^ M^−1^ s^−1^,^22^ leads one to estimate that the operator might remain unoccupied an average of 60 s. Moving at 10.3 bp/s, an RNA polymerase would traverse the near operator approximately 27 s after beginning transcription. If transcription does not begin immediately after adding NTP, then the possible time interval between the transit of an initially unoccupied operator by a TEC followed by LacI binding to the operator becomes very short. Consequently, a negligible fraction of initially unoccupied operator sites is expected to become filled after the transit of RNA polymerase within the 60 s time window before quenching.

## Does RNAP Wait for LacI to Dissociate or Does It Eject It?

Our results demonstrate that RNA polymerase halts but eventually bypasses LacI bound operator sites, especially the lower‐affinity O2 operator. The propensity of RNA polymerase to bypass LacI‐bound O2 is illustrated in Figure 3(A), which shows that RNA polymerase progress along the O2‐O1 sequence is only marginally affected by the addition of LacI. Three factors may contribute to the ability of RNA polymerase to bypass bound O2 operators: stochastic dissociation of LacI, RNA polymerase transcribing around the obstacle, and RNA polymerase actively dislodging LacI.

It is possible that RNA polymerase dislodges LacI from its operator. RNA polymerase II effectively dislodged nucleosomes from DNA templates and was more effective at higher transcriptional velocity.^2^ The SFM results reported here show that Os is a strong road block to transcription, even in the absence of looping [Fig. 3(A)]. The affinity of LacI for Os is about 20 times that for O2, meaning it is less likely to be dislodged by an advancing polymerase. Indeed, this is exhibited in the SFM results, in which only 10% of RNA polymerases pass a LacI bound to Os but 50% pass a LacI bound to O2 [Fig. 3(A)]. Magnetic tweezer transcription experiments utilizing DNA containing the stronger Os operator are currently in progress. Based on its dissociation rate, the occupancy lifetime of Os is expected to be roughly five times longer than O1. Consequently, changes in pause duration relative to the expected occupation time should reveal whether RNA polymerase is simply waiting for LacI to dissociate or actively dislodging the molecule.

The dissociation rates for LacI bound to DNA have been determined previously and suggest that in vivo LacI will dissociate after 2326, 434, or 102 s from Os, O1, and O2 respectively.^12^ For in vitro conditions similar to those described here, the O1 dissociation rate is reported to be 0.3/min^31^; the reciprocal suggests that LacI occupies an O1 operator for an average of 200 s. In the magnetic tweezer experiments pauses at the O1 site lasted an average of 246 s. This seems to indicate that in vitro, without accessory factors, RNA polymerase waits for LacI to dissociate.

There is also some evidence to suggest that RNA polymerase transcribes around LacI bound to O2. In several AFM images (Fig. 1), RNA polymerase appears to be in contact with the periphery of the DNA‐bound LacI. These images are qualitatively similar to what has been reported for RNA polymerase II transcribing through nucleosomes by loop diffusion.^2^ Based on this similarity, one might conclude that RNA polymerase bypasses LacI through a similar loop diffusion mechanism. However, it is important to note that, histone octamers exhibit lower affinity and sequence specificity for DNA and have a much larger footprint, both of which are thought to be essential for loop diffusion. By comparison, LacI is highly specific and has a smaller footprint meaning it is unlikely to be susceptible to loop diffusion. It is possible that LacI “steps” around RNA polymerase as it transcribes through the operator. In the open conformation, LacI can reach as far as 14 nm between its operator binding sites, a distance that is large enough to straddle the RNA polymerase to let it pass. This mechanism is also supported by the fact that RNA polymerase induces local bending in the DNA, which would serve to reduce the distance LacI would need to span.^32^

## Conclusion

Magnetic tweezers and scanning force microscopy assays showed several new features of the LacI protein. By limiting active transcription to a defined time interval, the rate of elongation was determined using scanning force microscopy. The disposition of halted RNA polymerases and LacI obstacles in nano‐topographs showed the expected dependence of the probability of progress on the operator strength for unlooped templates, but even LacI on a weak operator became an efficient roadblock to transcription elongation on a looped template. Thus, while the affinity of LacI for the binding site determines the strength of the roadblock in unlooped DNA constructs, it is not as critical for looped templates. Finally, the two conformations of LacI were clearly distinguished for the first time in both unlooped and looped complexes. It remains to be seen whether they constitute roadblocks of equivalent strength, but the clear images of the open LacI conformation suggested that the protein could straddle a TEC and therefore contribute to the mechanisms that resolve TEC halting by a LacI roadblock.

## Materials and Methods

### DNA for scanning force microscopy

1523 bp‐long DNA fragments were produced by PCR using plasmid templates, pYY_I1_400_BstEII or pZV_21_400 (Genbank format files in supplementary information), with an unlabeled forward primer and a biotin‐labeled reverse primer, and purified using a QIAQuick PCR Cleanup kit (Qiagen, Germantown, MD). The fragment contained the T7A1 promoter close to the upstream end, a “near” operator 261 bp downstream, a “far” operator 669 bp downstream, the lambda t1 terminator 1298 bp downstream, and a biotin‐label at the far downstream end for tagging with streptavidin (Fig. 1).

### Sample preparation for scanning force microscopy

As described in previous reports,^33^, ^34^ shortly before deposition of the sample, a 5 μl droplet of 0.01 ug/ml of poly‐L‐ornithine (1 kDa MW, Sigma‐Aldrich, St. Louis, MO) was deposited onto freshly cleaved mica and incubated for 2 min. The poly‐L‐ornithine‐coated mica was rinsed drop‐wise with 400 μl of high‐performance liquid chromatography grade water and dried with compressed air. Complexes of RNA polymerase bound at the promoter (PC) were produced by incubating 1 nM of DNA with 0.1 μM streptavidin, 7.5 nM LacI (unless otherwise stated) and RNA polymerase holoenzyme (New England Biolabs, Ipswitch, MA) diluted 200 times in transcription buffer (TXB; 20 mM Tris‐glutamate (pH 8.0), 10 mM magnesium‐glutamate, 50 mM potassium‐glutamate, 1mM DTT) for 30 min at 37**°**C. To initiate transcription, the reaction mixture was spiked with 1 mM NTPs to give a final concentration of 100 μM, and incubating at 37**°**C for 60 s. Elongation was terminated by spiking the mixture with 250 mM EDTA in TXB to give a final concentration of 20 mM EDTA, and incubating at 37**°**C for 30 s. 5 μl of the sample solution containing DNA and proteins were deposited on the poly‐L‐ornithine‐coated mica and incubated for 2 min. This droplet was rinsed with 400 μl of high‐performance liquid chromatography grade water and dried gently with compressed air.

### Scanning force microscopy and tracing DNA contours

Images were acquired with a NanoScope MultiMode VIII AFM microscope (Bruker Nano Surfaces, Santa Barbara, CA, USA) operating in Peak Force Tapping Mode using ScanAsyst‐Air cantilevers with 2 nm nominal tip radius. Areas of 5 × 5 μm^2^ were scanned at a rate of 0.27 Hz with a resolution of 2560 × 2560 pixels. After filtering the images to remove scan line offsets and tilt/bow, DNA molecules were interactively traced with the NeuronJ^35^ plug‐in of ImageJ.^36^ For both the looped and unlooped molecules, segments between end points and proteins, or between two proteins, were measured to determine the positions of all proteins along the DNA. Figure 3 and Supporting Information Fig. S2 show the ensemble of lac repressor positions for both looped and unlooped was tightly grouped at the near and far operator sites. The observed loops were between these two positions. The position of protein along each DNA molecule was normalized by the measured DNA length of the molecule set equal to 1524 bp.

### Magnetic tweezers

The real‐time transcription experiments were conducted using a custom‐built inverted microscope equipped with magnetic tweezers. The microscope is similar in construction to several previously described.^37^, ^38^, ^39^ Magnetic tweezers use a magnetic dipole to exert forces and torques on paramagnetic microspheres tethered to the bottom of a sample chamber by a DNA molecule (Supporting Information Fig. S5). The height of a tethered microsphere relative to the objective focal plane is measured by comparing the bright‐field diffraction pattern of the bead to a previously recorded lookup table containing bead‐diffraction patterns as a function of objective position. The length of the tether can be calculated by subtracting the relative height of the tethered particle from the relative height of a reference particle bound to the chamber surface (Supporting Information Fig. S5).

The microscope was assembled using a Nikon Plan 100x/1.25 Oil immersion objective (Nikon Instruments Inc. Melville, NY), P‐721 Piezo Flexure Objective Scanner (PI Physik Instrumente LP Auburn, MA), an *f* = 160 mm tube lens (Thorlabs Inc. Newton, NJ), and a Basler acA2000‐165um camera (IVS Imaging, Coppell, TX). Samples were illuminated using a custom LED (Luxeon Star LEDs, Quadica Developments Inc. Brantford, ON, Canada), brightfield illuminator. The magnetic dipole consisted of two 1/2**″**x1/4**″**X1/8**″** Neodymium N52 grade magnets (K&J Magnetics Inc. Pipersville, PA), spaced 1 mm apart, attached to a steel hub, mounted on a vertical translation and rotation stage (custom design) along the bright‐field beam path.

Real‐time 3D particle tracking was implemented following a previously published scheme.^40^ The *XY*‐location of each particle was tracked using a radial symmetry detection algorithm.^41^ The combination of objective, lens, and camera yielded a pixel resolution of 72.5 nm/pixel. With moderate image noise the radial symmetry algorithm localized particles to within 5–10% of a pixel, yielding an effective lateral accuracy of around 3–7 nm. *Z*‐positions were determined by matching the radial profile of diffraction pattern intensity ( ${\hat{I}}_{r}$) with the intensity pattern in the lookup table ( $I_{r}[z])$ that yielded the smallest total squared difference $\left(arg\text{min}{[\sum_{r}\left(I_{r}[z\right]-{\hat{I}}_{r})}^{2}]\right)$. For these experiments a finite sampled lookup table ( $I_{r}[z_{k}]$, with k = 1,2,…) was used. The sub‐step height was calculated by fitting the squared intensity differences to a parabola and using the vertex as the best estimate of *Z*. Calibration experiments revealed that this scheme yielded a depth resolution of 10‐20 nm. Microscope controls and 3D tracking software were written in MATLAB (Mathworks Natick, MA) and utilize Micro‐manager (www.micro-manager.org) to communicate with the hardware. Tracking routines and control software can be found at http://www.physics.emory.edu/faculty/finzi/research/code.shtml.

### DNA for MT

A 3025 bp DNA fragment was produced by PCR using a plasmid template, pYY_N1400_BstEII (Supplementary information F1), with an unlabeled forward primer and a biotin‐labeled reverse primer, and purified using a QIAQuick PCR Cleanup kit. The template contained the T7A1 promoter close to the upstream end, an O1 operator 689 bp downstream, the lambda t1 terminator 1298 bp downstream, and a biotin at the far, downstream end for attachment to a streptavidin‐coated bead [Fig. 4(A)]. The DNA template encoded only A, G, and U ribonucleotides until +22, in order to be able to stall the transcription elongation complex (TEC) by withholding CTP.

### Sample preparation for magnetic tweezers

Chambers were prepared as previously reported.^42^, ^43^ In brief, micro‐chambers with an approximate volume of ∼30 µL were prepared using a laser‐cut Parafilm gasket, which was heated to seal it between two coverslips (Fisherbrand, Thermo Fisher Scientific, Waltham, MA), cleaned with laboratory soap, rinsed with water, and stored in ethanol. The inlet and outlet channels were narrow to reduce evaporation of solution.^44^ The chamber was then incubated with 10 µg/mL purified Anti‐HA 11 Epitope tag antibody (16B12, monoclonal, Biolegend, San Diego, CA) in TXB (without magnesium glutamate) at 4˚C overnight (≤ 16 hrs) or at room temperature for 1 hr. Then, the surface was passivated with TXB (without magnesium glutamate) supplemented with 3 mg/mL α‐casein (Sigma‐Aldrich, St. Louis, MO) at room temperature for 1 h.

Stalled elongation complexes (SECs) were produced by incubating 25 nM doubly‐HA tagged *E. coli* RNA polymerase (Karen Adelman Laboratory, NIH), 10 nM DNA template, 50 µM GpA (initiating dinucleotide), and 10 µM ATP/UTP/GTP in TXB supplemented with 0.2 mg/mL α‐casein at 37˚C for 30 mins. SECs were then drawn into the chamber and incubated 30 min at room temperature to let the HA‐labeled RNA polymerase bind to the anti‐HA‐coated surface. The far end of the DNA from the promoter was then labeled with a 1.0 µm diameter, streptavidin‐coated paramagnetic bead (Dynabead MyOne Streptavidin T1, Invitrogen, Grand Island, NY) by incubating it with 20 µg/ml for 15 min. The experimental construct in the magnetic tweezers microscope is schematically illustrated in Supporting Information Figure S5. The extension of the DNA tether was monitored after introducing 1 mM NTPs with/without LacI (10 nM or 1 nM) in TXB supplemented with 0.2 mg/mL α‐casein.

### Acquisition and analysis of magnetic tweezer data

Extension‐versus‐time data were acquired at 164 Hz using a custom‐built instrument. The single biotin label at the end far from the T7A1 promoter acted as a swivel to torsionally relax the tether during transcription. Before adding NTPs, the extension of the tether was recorded for approximately 1 minute. Immediately after addition of 1 mM NTPs, turbulence lasting almost one minute produced spurious length measurements. When the turbulence subsided, many tethers returned to the previously measured extension value and shortly thereafter transcription elongation resumed and the DNA extension decreased.

A 60‐point moving average of the motions of beads that were stuck to the surface was used to subtract mechanical drift introduced by vibration or thermal expansion of the microscope. A 200‐point moving average of the drift‐corrected time‐series was applied to abate the noise in each time series. Pausing times were estimated as illustrated in Supporting Information Figure S6 by fitting sections of the time series with linear functions representing (1) transcription before pausing, (2) pausing, and (3) transcription after pausing. The duration of a pause was estimated as the distance between the intersections of lines 1 and 2 and lines 2 and 3.

## Supporting information

Supporting Information Figures.

Supporting Information.

Supporting Information.

## References

[1] Hodges C , Bintu L , Lubkowska L , Kashlev M , Bustamante C (2009) Nucleosomal fluctuations govern the transcription dynamics of RNA polymerase II. Science 325:626–628. 19644123 10.1126/science.1172926 PMC2775800

[2] Bintu L , Kopaczynska M , Hodges C , Lubkowska L , Kashlev M , Bustamante C (2011) The elongation rate of RNA polymerase determines the fate of transcribed nucleosomes. Nat Struct Mol Biol 18:1394–1399. 22081017 10.1038/nsmb.2164 PMC3279329

[3] Bintu L , Ishibashi T , Dangkulwanich M , Wu YY , Lubkowska L , Kashlev M , Bustamante C (2012) Nucleosomal elements that control the topography of the barrier to transcription. Cell 151:738–749. 23141536 10.1016/j.cell.2012.10.009 PMC3508686

[4] Jin J , Bai L , Johnson DS , Fulbright RM , Kireeva ML , Kashlev M , Wang MD (2010) Synergistic action of RNA polymerases in overcoming the nucleosomal barrier. Nat Struct Mol Biol 17:745–752. 20453861 10.1038/nsmb.1798 PMC2938954

[5] Zhang T , Cooper S , Brockdorff N (2015) The interplay of histone modifications – writers that read. EMBO Rep 16:1467–1481. 26474904 10.15252/embr.201540945 PMC4641500

[6] Long Hannah K , Prescott Sara L , Wysocka J (2016) Ever‐changing landscapes: transcriptional enhancers in development and evolution. Cell 167:1170–1187. 27863239 10.1016/j.cell.2016.09.018 PMC5123704

[7] Mendes MA , Guerra RF , Berns MC , Manzo C , Masiero S , Finzi L , Kater MM , Colombo L (2013) MADS domain transcription factors mediate short‐range DNA looping that is essential for target gene expression in Arabidopsis. Plant Cell 25:2560–2572. 23847151 10.1105/tpc.112.108688 PMC3753383

[8] Matthews KS (1992) DNA looping. Microbiol Rev 56:123–136. 1579106 10.1128/mr.56.1.123-136.1992 PMC372857

[9] Wilson CJ , Zhan H , Swint‐Kruse L , Matthews KS (2007) The lactose repressor system: paradigms for regulation, allosteric behavior and protein folding. Cell Mol Life Sci 64:3–16. 17103112 10.1007/s00018-006-6296-z PMC11136226

[10] Hsieh WT , Whitson PA , Matthews KS , Wells RD (1987) Influence of sequence and distance between two operators on interaction with the lac repressor. J Biol Chem 262:14583–14591. 3667591

[11] Pfahl M , Gulde V , Bourgeois S (1979) “Second” and “third operator” of the lac operon: an investigation of their role in the regulatory mechanism. J Mol Biol 127:339–344. 430569 10.1016/0022-2836(79)90333-4

[12] Garcia HG , Phillips R (2011) Quantitative dissection of the simple repression input‐output function. Proc Natl Acad Sci USA 108:12173–12178. 21730194 10.1073/pnas.1015616108 PMC3141941

[13] Sadler JR , Sasmor H , Betz JL (1983) A perfectly symmetric lac operator binds the lac repressor very tightly. Proc Natl Acad Sci USA 80:6785–6789. 6316325 10.1073/pnas.80.22.6785 PMC390070

[14] Oehler S , Müller‐Hill B (2010) High local concentration: a fundamental strategy of life. J Mol Biol 395:242–253. 19883663 10.1016/j.jmb.2009.10.056

[15] Whitson PA , Hsieh WT , Wells RD , Matthews KS (1987) Influence of supercoiling and sequence context on operator DNA‐binding with lac repressor. J Biol Chem 262:14592–14599. 3667592

[16] Oehler S , Amouyal M , Kolkhof P , von Wilcken‐Bergmann B , Muller‐Hill B (1994) Quality and position of the three lac operators of E. coli define efficiency of repression. EMBO J 13:3348–3355. 8045263 10.1002/j.1460-2075.1994.tb06637.x PMC395232

[17] Deuschle U , Gentz R , Bujard H (1986) lac repressor blocks transcribing RNA polymerase and terminates transcription. Proc Natl Acad Sci USA 83:4134–4137. 3520567 10.1073/pnas.83.12.4134 PMC323685

[18] Hao N , Krishna S , Ahlgren‐Berg A , Cutts EE , Shearwin KE , Dodd IB (2014) Road rules for traffic on DNA‐systematic analysis of transcriptional roadblocking in vivo. Nucleic Acids Res 42:8861–8872. 25034688 10.1093/nar/gku627 PMC4132739

[19] Deuschle U , Hipskind RA , Bujard H (1990) RNA polymerase II transcription blocked by Escherichia coli lac repressor. Science 248:480–483. 2158670 10.1126/science.2158670

[20] Reines D , Mote J Jr (1993) Elongation factor SII‐dependent transcription by RNA polymerase II through a sequence‐specific DNA‐binding protein. Proc Natl Acad Sci USA 90:1917–1921. 8446609 10.1073/pnas.90.5.1917 PMC45991

[21] Arndt KM , Chamberlin MJ (1990) RNA chain elongation by Escherichia coli RNA polymerase ‐ factors affecting the stability of elongating ternary complexes. J Mol Biol 213:79–108. 1692594 10.1016/S0022-2836(05)80123-8

[22] Wong OK , Guthold M , Erie DA , Gelles J (2008) Interconvertible lac repressor‐DNA loops revealed by single‐molecule experiments. PLoS Biol 6:e232. 18828671 10.1371/journal.pbio.0060232 PMC2553838

[23] Rutkauskas D , Zhan H , Matthews KS , Pavone FS , Vanzi F (2009) Tetramer opening in LacI‐mediated DNA looping. Proc Natl Acad Sci USA 106:16627–16632. 19805348 10.1073/pnas.0904617106 PMC2757816

[24] Davenport RJ , Wuite GJ , Landick R , Bustamante C (2000) Single‐molecule study of transcriptional pausing and arrest by E. coli RNA polymerase. Science 287:2497–2500. 10741971 10.1126/science.287.5462.2497

[25] Neuman KC , Abbondanzieri EA , Landick R , Gelles J , Block SM (2003) Ubiquitous transcriptional pausing is independent of RNA polymerase backtracking. Cell 115:437–447. 14622598 10.1016/s0092-8674(03)00845-6

[26] Schafer DA , Gelles J , Sheetz MP , Landick R (1991) Transcription by single molecules of RNA polymerase observed by light microscopy. Nature 352:444–448. 1861724 10.1038/352444a0

[27] Johnson S , Lindén M , Phillips R (2012) Sequence dependence of transcription factor‐mediated DNA looping. Nucleic Acids Res 40:7728–7738. 22718983 10.1093/nar/gks473 PMC3439888

[28] Forde NR , Izhaky D , Woodcock GR , Wuite GJ , Bustamante C (2002) Using mechanical force to probe the mechanism of pausing and arrest during continuous elongation by Escherichia coli RNA polymerase. Proc Natl Acad Sci USA 99:11682–11687. 12193647 10.1073/pnas.142417799 PMC129329

[29] Levandoski MM , Tsodikov OV , Frank DE , Melcher SE , Saecker RM , Record Jr TM (1996) Cooperative and anticooperative effects in binding of the first and second plasmid OsymOperators to a LacI tetramer: evidence for contributions of non‐operator DNA binding by wrapping and looping. J Mol Biol 260:697–717. 8709149 10.1006/jmbi.1996.0431

[30] Priest DG , Cui L , Kumar S , Dunlap DD , Dodd IB , Shearwin KE (2014) Quantitation of the DNA tethering effect in long‐range DNA looping in vivo and in vitro using the Lac and λ repressors. Proc Natl Acad Sci USA 111:349–354. 24344307 10.1073/pnas.1317817111 PMC3890862

[31] Johnson S , van de Meent JW , Phillips R , Wiggins CH , Linden M (2014) Multiple LacI‐mediated loops revealed by Bayesian statistics and tethered particle motion. Nucleic Acids Res 42:10265–10277. 25120267 10.1093/nar/gku563 PMC4176382

[32] Ruff E , Record M , Artsimovitch I (2015) Initial events in bacterial transcription initiation. Biomolecules 5:1035. 26023916 10.3390/biom5021035 PMC4496709

[33] Wang H , Finzi L , Lewis DE , Dunlap D (2009) AFM studies of lambda repressor oligomers securing DNA loops. Curr Pharm Biotechnol 10:494–501. 19689317 10.2174/138920109788922155 PMC3641193

[34] Wang H , Dodd IB , Dunlap DD , Shearwin KE , Finzi L (2013) Single molecule analysis of DNA wrapping and looping by a circular 14mer wheel of the bacteriophage 186 CI repressor. Nucleic Acids Res 41:5746–5756. 23620280 10.1093/nar/gkt298 PMC3675496

[35] Meijering E , Jacob M , Sarria JC , Steiner P , Hirling H , Unser M (2004) Design and validation of a tool for neurite tracing and analysis in fluorescence microscopy images. Cytometry A58:167–176. 10.1002/cyto.a.20022 15057970

[36] Schneider CA , Rasband WS , Eliceiri KW (2012) NIH Image to ImageJ: 25 years of image analysis. Nat Meth 9:671–675. 10.1038/nmeth.2089 PMC5554542 22930834

[37] Ribeck N , Saleh OA (2008) Multiplexed single‐molecule measurements with magnetic tweezers. Rev Sci Instrum 79:094301. 19044437 10.1063/1.2981687

[38] Seol Y , Neuman KC (2011) Magnetic tweezers for single‐molecule manipulation. Methods Mol Biol 783:265–293. 21909894 10.1007/978-1-61779-282-3_15

[39] Ding Y , Manzo C , Fulcrand G , Leng F , Dunlap D , Finzi L (2014) DNA supercoiling: a regulatory signal for the λ repressor. Proc Natl Acad Sci USA 111:15402–15407. 25319264 10.1073/pnas.1320644111 PMC4217475

[40] Gosse C , Croquette V (2002) Magnetic tweezers: micromanipulation and force measurement at the molecular level. Biophys J 82:3314–3329. 12023254 10.1016/S0006-3495(02)75672-5 PMC1302119

[41] Parthasarathy R (2012) Rapid, accurate particle tracking by calculation of radial symmetry centers. Nat Methods 9:724–726. 22688415 10.1038/nmeth.2071

[42] Kumar S , Manzo C , Zurla C , Ucuncuoglu S , Finzi L , Dunlap D (2014) Enhanced tethered‐particle motion analysis reveals viscous effects. Biophys J 106:399–409. 24461015 10.1016/j.bpj.2013.11.4501 PMC3907251

[43] Priest DG , Kumar S , Yan Y , Dunlap DD , Dodd IB , Shearwin KE (2014) Quantitation of interactions between two DNA loops demonstrates loop domain insulation in E. coli cells. Proc Natl Acad Sci USA 111:E4449–44E4457. 25288735 10.1073/pnas.1410764111 PMC4210295

[44] Kovari DT , Yan Y , Dunlap D , Finzi L (2017) Tethered particle motion: an easy technique for probing DNA topology and transcription factor interactions. Methods Mol Biol in press. 10.1007/978-1-4939-7271-5_17 PMC6089228 28940077

