# Supplementary material for: Proteins mediating DNA loops effectively block transcription
Source: Protein Sci. 2017 Mar 27;26(7):1427–38. doi: 10.1002/pro.3156 (PMC5477534; doi:10.1002/pro.3156)
Supplement: Supplementary file 1 — Supporting Information Figures. [file PRO-26-1427-s001.pdf]

Figure S1

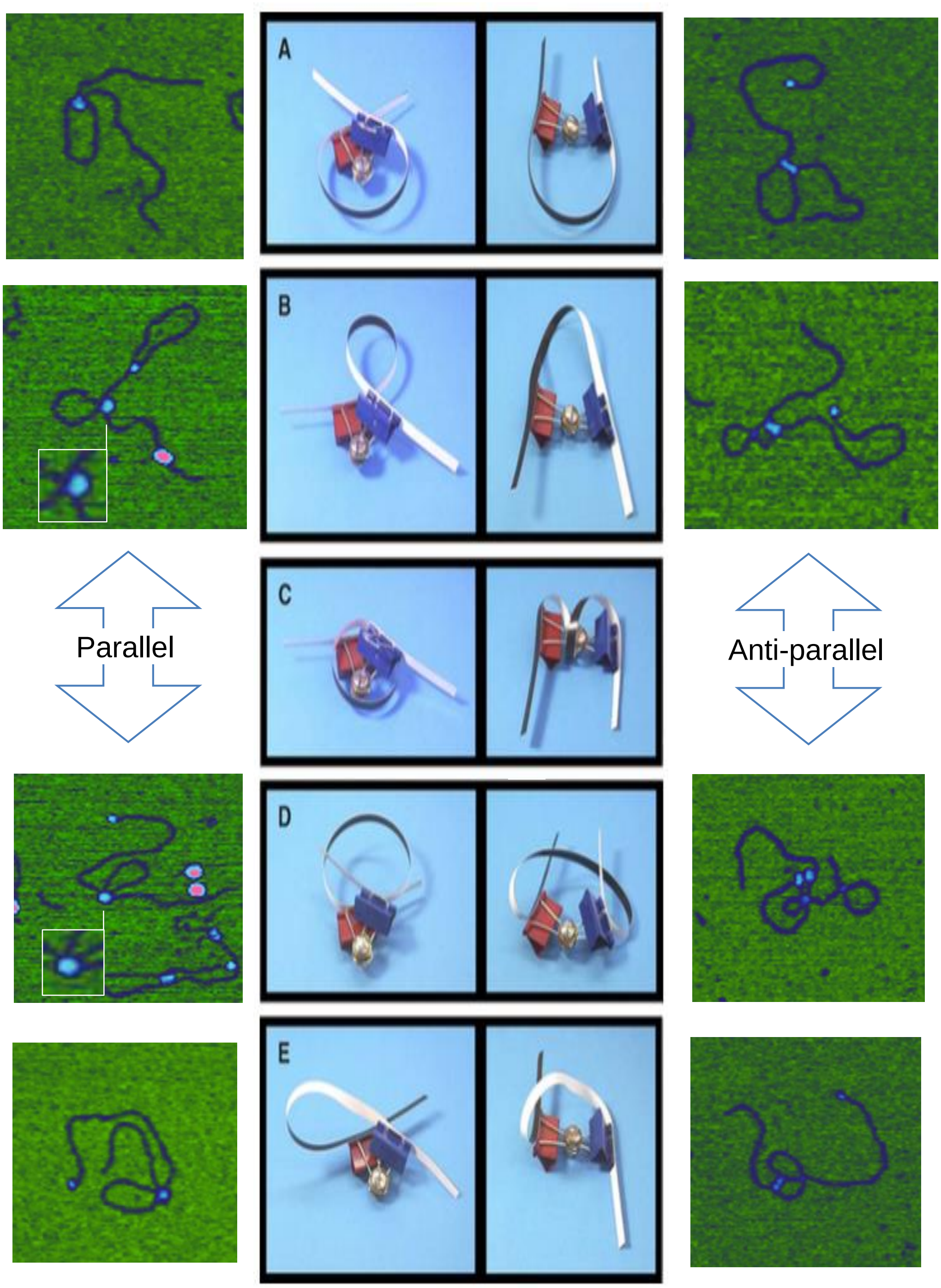

Figure S1. Several configurations of LacI-mediated loops were observed in SFM images (lateral). They had been previously speculated to exist as diagramed in the figures (central) from Wong et al. (Wong, O. K., et al. (2008). "Interconvertible lac repressor-DNA loops revealed by single-molecule experiments." PLoS Biol 6(9): e232).

Figure S2

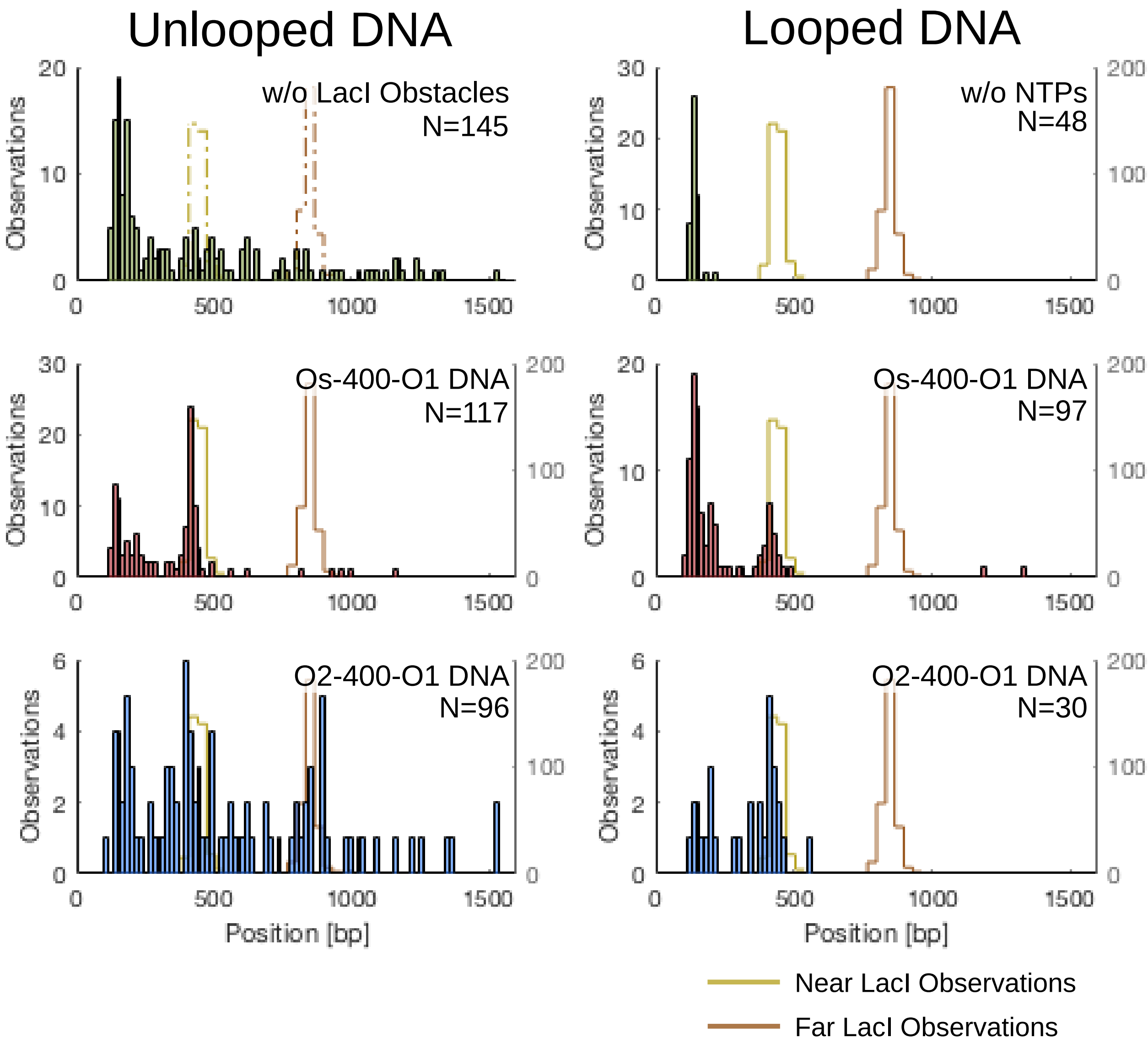

Figure S2. Histograms of RNAP positions. The positons of RNA polymerase and LacI molecules were measured by tracing along the contour of the Os-400-O1- or O2-400-O1-containing DNA molecules in SFM images. In all plots, left axis reports frequency of RNA polymerase observations; right axis reports frequency of LacI observations. Experiments were performed with and without NTP and/or LacI as indicated. DNA molecules were categorized into either unlooped (left column) or looped (right column) topologies. (upper right) Without NTPs, RNAP remained at the promoter. (upper left) With NTP, but with no LacI present to act as a barrier, RNAP transcribes the entire gene. The probability that RNAP transcribes a given length decays roughly exponentially. (middle row) Os is a strong roadblock to transcription; for both unlooped (left) and looped (right) DNA nearly all RNAP molecules were observed before or halted at the near operator (yellow). (lower left) Without looping, O2 is a weak barrier to transcription. A significant number of molecules were observed beyond the near operator, or stalled at the far operator (orange histogram). (lower right) Looping appears to stabilize O2 as a barrier to transcription. Only one molecule was observed past the near operator.

Figure S3

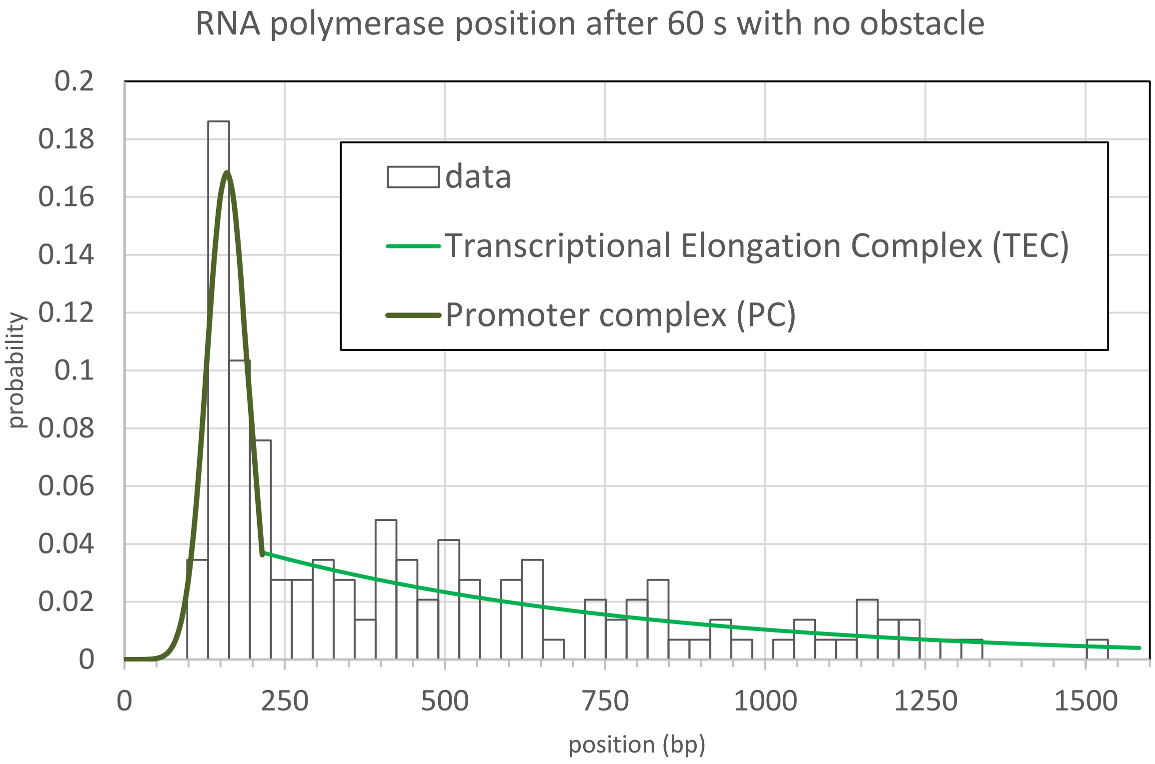

Figure S3. The distribution of positions of RNA polymerase after 60 s of transcription with no obstacles (145 molecules). The histogram was fit with a Gaussian corresponding to the inactive complexes that remained at the promoter (dark green) and a decaying exponential corresponding to the active transcriptional elongation complexes that advanced along the DNA molecules. The decay constant of the fitted exponential was 660 bp.

Figure S4

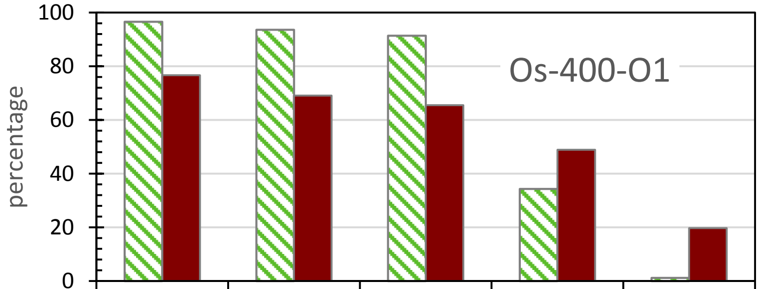

	LacI at Os	LacI at O1	LacI at Both	Looped	No LacI
- NTP	96.6	93.6	91.4	34.3	1.2
+ NTP	76.7	69.1	65.5	48.9	19.7

N=501

N=1175

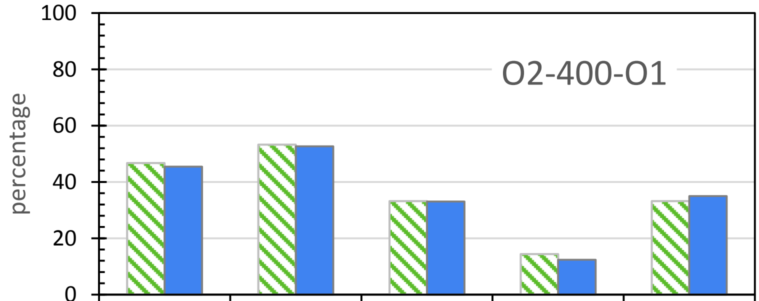

	LacI at O2	LacI at O1	LacI at Both	Looped	No LacI
- NTP	46.7	53.3	33.2	14.4	33.2
+ NTP	45.4	52.7	33.1	12.4	35.0

N=458

N=526

Figure S4. Active transcription did not diminish the fraction of looped molecules. In SFM images only molecules with unambiguous topologies with or without bound RNAP were selected for analysis. **a)** In Os-400-O1 molecules, operators were nearly saturated with LacI without NTPs (dark green stripe) and decreased roughly 20% with NTP (solid red). However, the fraction of looped molecules increased with active transcription. **b)** In contrast, roughly half of operators O2-400-O1 molecules were occupied by LacI without NTP (light green stripe) and the fraction did not change with NTP (solid blue). Active transcription negligibly changed the fraction of looped molecules. Numbers of molecules categorized were 501 and 1175 for Os-400-O1 with and without NTP, and 458 and 526 for O2-400-O1 with or without NTP. Note that “LacI at Os” (or “LacI at O2”) and LacI at O1” and “LacI at both” are not exclusive categories.

Figure S5

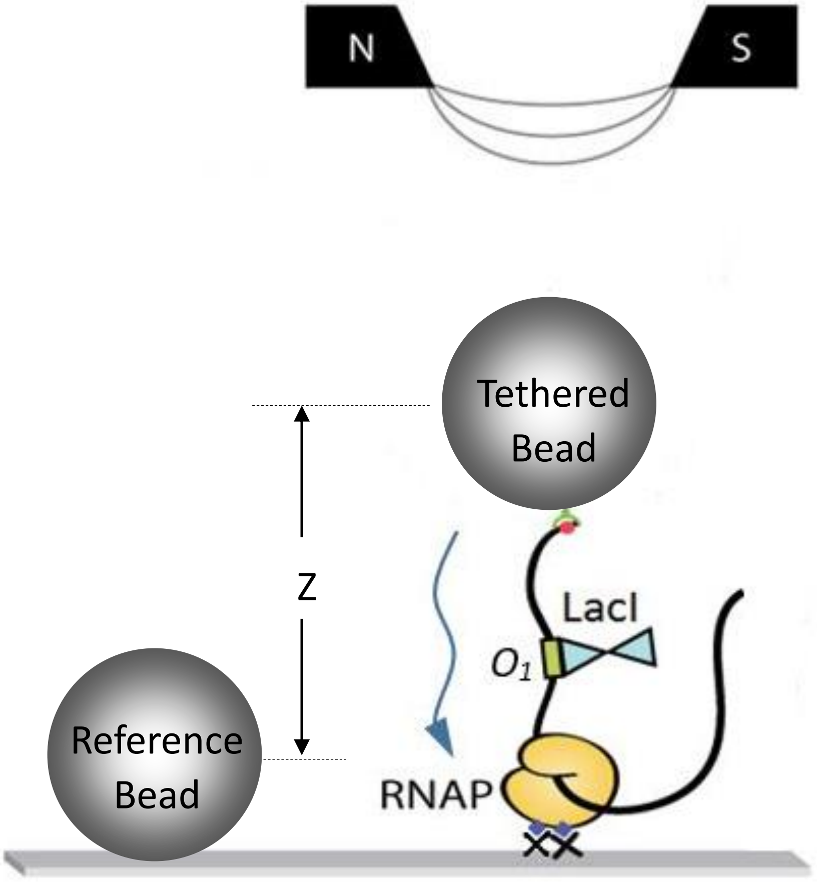

Figure S5. A schematic diagram of the magnetic tweezer transcription assay. The DNA tether is attached to the bead through a streptavidin-biotin linkage (green-red), and RNAP (yellow) is attached to the surface through linkages between two HA tags on the enzyme and the anti-HA-coated glass surface of the microchamber. The tether includes an O1 operator to which lac repressor may bind. Transcriptional activity will shorten the DNA tether as RNA polymerase draws the DNA template through itself.

Figure S6

1) Fitting the rate of DNA extension decrease before pausing, yields: y1=-0.001276x+0.1831

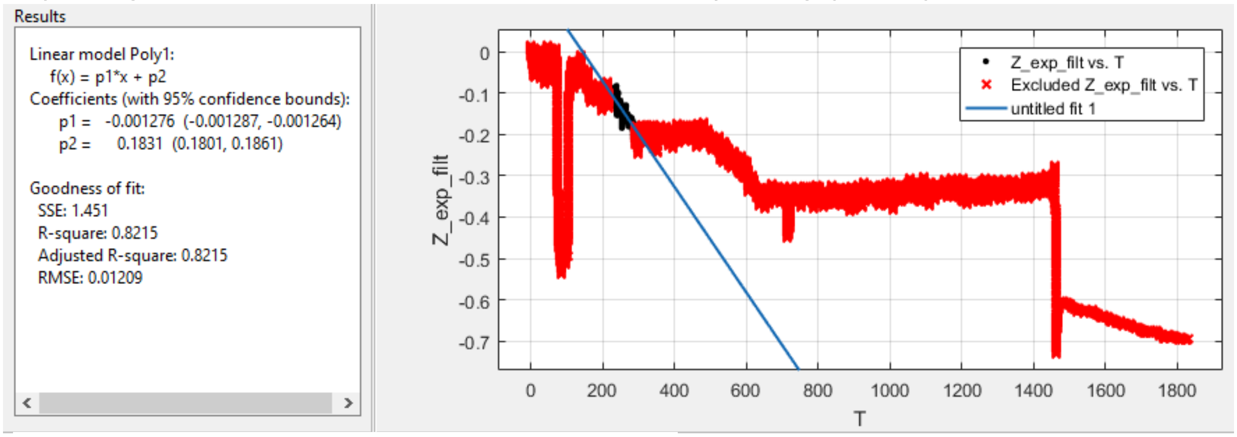

2) Fitting the pause duration, yields: y2=-0.2058

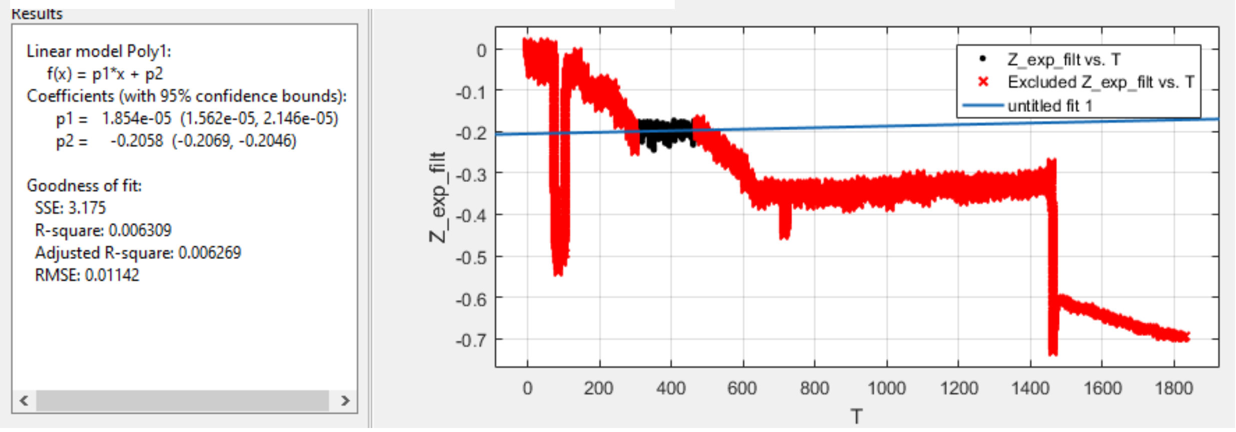

3) Fitting the rate of DNA extension decrease before pausing, yields: y3= -0.0009094+0.2479

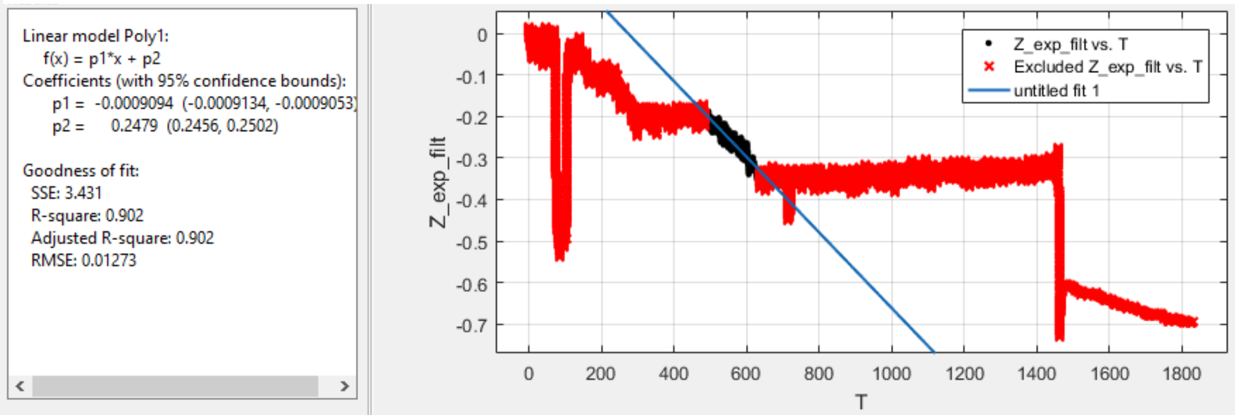

Figure S6. A graphic illustration of the method used to calculate the duration of an RNAP pause. The time at which the pause begins is given by the intersection between y1 and y2, -0.001276x+0.1831=-0.2058 → t_i=304 s. The time at which the pause ends is given by intersection of y2 and y3, -0.2058= -0.0009094+0.2479 → t_t=498 s. The duration of the pause due to LacI bound O1 operator is then, T= t_t- t_i=194 s
