# Supplementary material for: Proteins mediating DNA loops effectively block transcription
Source: Protein Sci. 2017 Mar 27;26(7):1427–38. doi: 10.1002/pro.3156 (PMC5477534; doi:10.1002/pro.3156)
Supplement: Supplementary file 2 — Supporting Information. [file PRO-26-1427-s002.pdf]

```
LOCUS       pYY_I1_400_BstEI        5847 bp ds-DNA     circular     16-
DEC-2016
DEFINITION  .
ACCESSION
VERSION
SOURCE      .
  ORGANISM  .
COMMENT
COMMENT     ApEinfo:methylated:1
FEATURES             Location/Qualifiers
     misc_feature    3225..3696
                     /label=Fragment 5
                     /ApEinfo_fwdcolor=#ff80ff
                     /ApEinfo_revcolor=#ff80ff
                     /ApEinfo_graphicformat=arrow_data {{0 1 2 0 0 -1} {}
0}
                     width 5 offset 0
     misc_feature    2947..3254
                     /label=Fragment 4
                     /ApEinfo_fwdcolor=#80ffff
                     /ApEinfo_revcolor=#80ffff
                     /ApEinfo_graphicformat=arrow_data {{0 1 2 0 0 -1} {}
0}
                     width 5 offset 0
     misc_feature    2620..2976
                     /label=Fragment 3
                     /ApEinfo_fwdcolor=#80ff80
                     /ApEinfo_revcolor=#80ff80
                     /ApEinfo_graphicformat=arrow_data {{0 1 2 0 0 -1} {}
0}
                     width 5 offset 0
     misc_feature    join(2456..2591,2612..2649)
                     /label=Fragment 2
                     /ApEinfo_fwdcolor=#27a9ef
                     /ApEinfo_revcolor=#27a9ef
                     /ApEinfo_graphicformat=arrow_data {{0 1 2 0 0 -1} {}
0}
                     width 5 offset 0
     misc_feature    2151..2485
                     /label=Fragment 1
                     /ApEinfo_fwdcolor=#cca633
                     /ApEinfo_revcolor=#cca633
                     /ApEinfo_graphicformat=arrow_data {{0 1 2 0 0 -1} {}
0}
                     width 5 offset 0
     CDS             complement(4735..5394)
                     /label=AmpR
                     /ApEinfo_fwdcolor=yellow
                     /ApEinfo_revcolor=yellow
                     /ApEinfo_graphicformat=arrow_data {{0 1 2 0 0 -1} {}
0}
                     width 5 offset 0
     rep_origin      complement(3955..4637)
                     /label=ColE1 origin
```

```
0}
                     /ApEinfo_fwdcolor=gray50
                     /ApEinfo_revcolor=gray50
                     /ApEinfo_graphicformat=arrow_data {{0 1 2 0 0 -1} {}
0}
                     width 5 offset 0
     misc_feature    2990..3010
                     /label=lac O1
                     /ApEinfo_fwdcolor=#ff8040
                     /ApEinfo_revcolor=#ff8040
                     /ApEinfo_graphicformat=arrow_data {{0 1 2 0 0 -1} {}
0}
                     width 5 offset 0
     misc_feature    2592..2611
                     /label=Fragment 2(1)
                     /ApEinfo_label=Fragment 2
                     /ApEinfo_fwdcolor=#8bb3f3
                     /ApEinfo_revcolor=#8bb3f3
                     /ApEinfo_graphicformat=arrow_data {{0 1 2 0 0 -1} {}
0}
                     width 5 offset 0
     red             3602..3654
                     /label=Lambda t1 terminator
                     /ApEinfo_fwdcolor=red
                     /ApEinfo_revcolor=green
                     /ApEinfo_graphicformat=arrow_data {{0 1 2 0 0 -1} {}
0}
                     width 5 offset 0
     misc_feature    2346..2352
                     /label=BstEII
                     /ApEinfo_fwdcolor=#ff80c0
                     /ApEinfo_revcolor=green
                     /ApEinfo_graphicformat=arrow_data {{0 1 2 0 0 -1} {}
0}
                     width 5 offset 0
     protein_bind    2592..2611
                     /label=Lac Oid
                     /ApEinfo_fwdcolor=#c3063c
                     /ApEinfo_revcolor=green
                     /ApEinfo_graphicformat=arrow_data {{0 1 2 0 0 -1} {}
0}
                     width 5 offset 0
     misc_feature    2351..2351
                     /label=stall site +22
                     /ApEinfo_fwdcolor=#f5010a
                     /ApEinfo_revcolor=green
                     /ApEinfo_graphicformat=arrow_data {{0 1 2 0 0 -1} {}
0}
                     width 5 offset 0
     misc_feature    2086..2105
                     /label=S/JBOIDO1_400/2086
                     /ApEinfo_fwdcolor=cyan
                     /ApEinfo_revcolor=green
                     /ApEinfo_graphicformat=arrow_data {{0 1 2 0 0 -1} {}
0}
```

```
                     width 5 offset 0
     misc_feature    complement(5095..5111)
                     /label=A/JBOIDO1_400/5096_Apa1
                     /ApEinfo_fwdcolor=cyan
                     /ApEinfo_revcolor=green
                     /ApEinfo_graphicformat=arrow_data {{0 1 2 0 0 -1} {}
0}
                     width 5 offset 0
     misc_feature    2330..2331
                     /label=start point
                     /ApEinfo_fwdcolor=#ff0000
                     /ApEinfo_revcolor=green
                     /ApEinfo_graphicformat=arrow_data {{0 1 2 0 0 -1} {}
0}
                     width 5 offset 0
     promoter        2277..2330
                     /label=T7A1
                     /ApEinfo_fwdcolor=#bbffbb
                     /ApEinfo_revcolor=#c1ffff
                     /ApEinfo_graphicformat=arrow_data {{0 1 2 0 0 -1} {}
0}
                     width 5 offset 0
     misc_feature    2318..2323
                     /label=TATA Box
                     /ApEinfo_fwdcolor=#ff8000
                     /ApEinfo_revcolor=green
                     /ApEinfo_graphicformat=arrow_data {{0 1 2 0 0 -1} {}
0}
                     width 5 offset 0
ORIGIN
        1 tatcacagtt aaattgctaa cgcagtcagg caccgtgtat gaaatctaac
aatgcgctca
       61 tcgtcatcct cggcaccgtc accctggatg ctgtaggcat aggcttggtt
atgccggtac
      121 tgccgggcct cttgcgggat atcgtccatt ccgacagcat cgccagtcac
tatggcgtgc
      181 tgctagcgct atatgcgttg atgcaatttc tatgcgcacc cgttctcgga
gcactgtccg
      241 accgctttgg ccgccgccca gtcctgctcg cttcgctact tggagccact
atcgactacg
      301 cgatcatggc gaccacaccc gtcctgtgga tcctctacgc cggacgcatc
gtggccggca
      361 tcaccggcgc cacaggtgcg gttgctggcg cctatatcgc cgacatcacc
gatggggaag
      421 atcgggctcg ccacttcggg ctcatgagcg cttgtttcgg cgtgggtatg
gtggcaggcc
      481 ccgtggccgg gggactgttg ggcgccatct ccttgcatgc accattcctt
gcggcggcgg
      541 tgctcaacgg cctcaaccta ctactgggct gcttcctaat gcaggagtcg
cataagggag
      601 agcgtcgacc gatgcccttg agagccttca acccagtcag ctccttccgg
tgggcgcggg
      661 gcatgactat cgtcgccgca cttatgactg tcttctttat catgcaactc
gtaggacagg
```

721 tgccggcagc gctctgggtc attttcggcg aggaccgctt tcgctggagc gcgacgatga
781 tcggcctgtc gcttgcggta ttcggaatct tgcacgccct cgctcaagcc ttcgtcactg
841 gtcccgccac caaacgtttc ggcgagaagc aggccattat cgccggcatg gcggccgacg
901 cgctgggcta cgtcttgctg gcgttcgcga cgcgaggctg gatggccttc cccattatga
961 ttcttctcgc ttccggcggc atcgggatgc ccgcgttgca ggccatgctg tccaggcagg
1021 tagatgacga ccatcaggga cagcttcaag gatcgctcgc ggctcttacc agcctaactt
1081 cgatcattgg accgctgatc gtcacggcga tttatgccgc ctcggcgagc acatggaacg
1141 ggttggcatg gattgtaggc gccgccctat accttgtctg cctccccgcg ttgcgtcgcg
1201 gtgcatggag ccgggccacc tcgacctgaa tggaagccgg cggcacctcg ctaacggatt
1261 caccactcca agaattggag ccaatcaatt cttgcggaga actgtgaatg cgcaaaccaa
1321 cccttggcag aacatatcca tcgcgtccgc catctccagc agccgcacgc ggcgcatctc
1381 gggcagcgtt gggtcctggc cacgggtgcg catgatcgtg ctcctgtcgt tgaggacccg
1441 gctaggctgg cggggttgcc ttactggtta gcagaatgaa tcaccgatac gcgagcgaac
1501 gtgaagcgac tgctgctgca aaacgtctgc gacctgagca acaacatgaa tggtcttcgg
1561 tttccgtgtt tcgtaaagtc tggaaacgcg gaagtcagcg ccctgcacca ttatgttccg
1621 gatctgcatc gcaggatgct gctggctacc ctgtggaaca cctacatctg tattaacgaa
1681 gcgctggcat tgaccctgag tgatttttct ctggtcccgc cgcatccata ccgccagttg
1741 tttaccctca caacgttcca gtaaccgggc atgttcatca tcagtaaccc gtatcgtgag
1801 catcctctct cgtttcatcg gtatcattac ccccatgaac agaaatcccc cttacacgga
1861 ggcatcagtg accaaacagg aaaaaaccgc ccttaacatg gcccgcttta tcagaagcca
1921 gacattaacg cttctggaga aactcaacga gctggacgcg gatgaacagg cagacatctg
1981 tgaatcgctt cacgaccacg ctgatgagct ttaccgcagc tgcctcgcgc gtttcggtga
2041 tgacggtgaa aacctctgac acatgcagct cccggagacg gtcacagctt gtctgtaagc
2101 ggatgccggg agcagacaag cccgtcaggg cgcgtcagcg ggtgttggcA ggtgtcgggg
2161 cgcagccatg acccagtcac ccatggtgc agtatgaagg cggcggagcc gacaccacgg
2221 ccaccgatat tatttgcccg atgtacgcgc gcgtggatga agaccagccc ttcccggctt
2281 tatcaaaaag agtattgact taaagtctaa cctataggat acttacagcg atggagaggt

2341 gtagtggtaa ccagaagata agatggcttt cgctacctgg agagacgcgc
ccgctgatcc
2401 tttgcgaata cgcccacgcg atgggtaaca gtcttggcgg tttcgctaaa
tactggcagg
2461 cgtttcgtca gtatccccgt ttacagggcg gcttcgtctg ggactgggtg
gatcagtcgc
2521 tgattaaata tgatgaaaac ggcaacccgt ggtaccggct tacggcggtg
attttTgcga
2581 tacgccgaac gaattgtgag cgctcacaat tatcgccagt tctgtatgaa
cggtctggtc
2641 tttgccgacc gcacgccgca tccagcgctg acggaagcaa aacaccagca
gcagtttttc
2701 agttccgttt atccgggcaa accatcgaag tgaccagcga atacctgttc
cgtcatagcg
2761 ataacgagct cctgcactgg atggtggcgc tggatggtaa gccgctggca
agcggtgaag
2821 tgcctctgga tgtcgctcca caaggtaaac agttgattga actgTctgaa
ctaccgcagc
2881 cggagagcgc cgggcaactc tggctcacag tacgcgtagt gcaaccgaac
gcgaccgcat
2941 ggtcagaagc cgggcacatc agcgcctggc agcagtAgcg tctggcggaa
attgtgagcg
3001 gataacaatt aaacctcagt gtgacgctcc ccgccgcgtc ccacgccatc
ccgcatctga
3061 ccaccagcga aatggatttt tgcactcgag ctgggtaata agcgttggca
atttaaccgc
3121 cagtcaggct ttctttcaca gatgtggatt ggcgataaaa aacaactgct
gacgccgctg
3181 cgcgatcagt tcacccgtgc accgctggat aacgacattg gcgtaagtga
agcgacccgc
3241 attgacccta acgcctgggt cgaacActgg aaggcggcgg gccattacca
ggccgaagca
3301 gcgttgttgc agtgcacggc agatacactt gctgatgcgg tgctgattac
gaccgctcac
3361 gcgtggcagc atcaggggaa aaccttattt atcagccgga aaacctaccg
gattgatggt
3421 agtggtcaaa tggcgattac cgttgatgtt gaagtggcga gcgatacacc
gcatccggcg
3481 cggattggcc tgaactgcca gctggcgcag gtagcagagc gggtaaactg
gctcggatta
3541 gcggccgcaa gaaaactatc ccgaccgcct tactgccgcc tgttttgacc
gctgggatct
3601 gctgtaacag agcattagcg caaggtgatt tttgtcttct tgcgctaatt
ttttccattg
3661 tctagagtag cgatagcgga gtgtatactg gcttaactat gcggcatcag
agcagattgt
3721 actgagagtg caccatatgc ggtgtgaaat accgcacaga tgcgtaagga
gaaaataccg
3781 catcaggcgc tcttccgctt cctcgctcac tgactcgctg cgctcggtcg
ttcggctgcg
3841 gcgagcggta tcagctcact caaaggcggt aatacggtta tccacagaat
caggggataa
3901 cgcaggaaag aacatgtgag caaaaggcca gcaaaaggcc aggaaccgta
aaaaggccgc

3961 gttgctggcg tttttccata ggctccgccc ccctgacgag catcacaaaa
atcgacgctc
4021 aagtcagagg tggcgaaacc cgacaggact ataaagatac caggcgtttc
cccctggaag
4081 ctccctcgtg cgctctcctg ttccgaccct gccgcttacc ggatacctgt
ccgcctttct
4141 cccttcggga agcgtggcgc tttctcatag ctcacgctgt aggtatctca
gttcggtgta
4201 ggtcgttcgc tccaagctgg gctgtgtgca cgaacccccc gttcagcccg
accgctgcgc
4261 cttatccggt aactatcgtc ttgagtccaa cccggtaaga cacgacttat
cgccactggc
4321 agcagccact ggtaacagga ttagcagagc gaggtatgta ggcggtgcta
cagagttctt
4381 gaagtggtgg cctaactacg gctacactag aaggacagta tttggtatct
gcgctctgct
4441 gaagccagtt accttcggaa aaagagttgg tagctcttga tccggcaaac
aaaccaccgc
4501 tggtagcggt ggtttttttg tttgcaagca gcagattacg cgcagaaaaa
aaggatctca
4561 agaagatcct ttgatctttt ctacggggtc tgacgctcag tggaacgaaa
actcacgtta
4621 agggattttg gtcatgagat tatcaaaaag gatcttcacc tagatccttt
taaattaaaa
4681 atgaagtttt aaatcaatct aaagtatata tgagtaaact tggtctgaca
gttaccaatg
4741 cttaatcagt gaggcaccta tctcagcgat ctgtctattt cgttcatcca
tagttgcctg
4801 actccccgtc gtgtagataa ctacgatacg ggagggctta ccatctggcc
ccagtgctgc
4861 aatgataccg cgagacccac gctcaccggc tccagattta tcagcaataa
accagccagc
4921 cggaagggcc gagcgcagaa gtggtcctgc aactttatcc gcctccatcc
agtctattaa
4981 ttgttgccgg gaagctagag taagtagttc gccagttaat agtttgcgca
acgttgttgc
5041 cattgctgca ggcatcgtgg tgtcacgctc gtcgtttggt atggcttcat
tcagctccgg
5101 ttcccaacga tcaaggcgag ttacatgatc ccccatgttg tgcaaaaaag
cggttagctc
5161 cttcggtcct ccgatcgttg tcagaagtaa gttggccgca gtgttatcac
tcatggttat
5221 ggcagcactg cataattctc ttactgtcat gccatccgta agatgctttt
ctgtgactgg
5281 tgagtactca accaagtcat tctgagaata gtgtatgcgg cgaccgagtt
gctcttgccc
5341 ggcgtcaaca cgggataata ccgcgccaca tagcagaact ttaaaagtgc
tcatcattgg
5401 aaaacgttct tcggggcgaa aactctcaag gatcttaccg ctgttgagat
ccagttcgat
5461 gtaacccact cgtgcaccca actgatcttc agcatctttt actttcacca
gcgtttctgg
5521 gtgagcaaaa acaggaaggc aaaatgccgc aaaaaaggga ataagggcga
cacggaaatg

```
     5581 ttgaatactc atactcttcc tttttcaata ttattgaagc atttatcagg
gttattgtct
     5641 catgagcgga tacatatttg aatgtattta gaaaaataaa caaatagggg
ttccgcgcac
     5701 atttccccga aaagtgccac ctgacgtcta agaaaccatt attatcatga
cattaaccta
     5761 taaaaatagg cgtatcacga ggccctttcg tcttcaagaa ttctcatgtt
tgacagctta
     5821 tcatcgataa gctttaatgc ggtagtt
//
```
