# Supplementary material for: Proteins mediating DNA loops effectively block transcription
Source: Protein Sci. 2017 Mar 27;26(7):1427–38. doi: 10.1002/pro.3156 (PMC5477534; doi:10.1002/pro.3156)
Supplement: Supplementary file 3 — Supporting Information. [file PRO-26-1427-s003.pdf]

```
LOCUS       pZV_21_400              5848 bp ds-DNA     circular     09-
NOV-2016
DEFINITION  .
ACCESSION
VERSION
SOURCE      .
  ORGANISM  .
COMMENT
COMMENT     ApEinfo:methylated:1
FEATURES             Location/Qualifiers
     misc_feature    2948..3294
                     /label=Fragment 5
                     /ApEinfo_fwdcolor=#acffff
                     /ApEinfo_revcolor=#acffff
                     /ApEinfo_graphicformat=arrow_data {{0 1 2 0 0 -1} {}
0}
                     width 5 offset 0
     misc_feature    3226..3697
                     /label=Fragment 5(1)
                     /ApEinfo_label=Fragment 5
                     /ApEinfo_fwdcolor=#ff80ff
                     /ApEinfo_revcolor=#ff80ff
                     /ApEinfo_graphicformat=arrow_data {{0 1 2 0 0 -1} {}
0}
                     width 5 offset 0
     misc_feature    2621..2977
                     /label=Fragment 3
                     /ApEinfo_fwdcolor=#80ff80
                     /ApEinfo_revcolor=#80ff80
                     /ApEinfo_graphicformat=arrow_data {{0 1 2 0 0 -1} {}
0}
                     width 5 offset 0
     misc_feature    join(2456..2591,2613..2650)
                     /label=Fragment 2
                     /ApEinfo_fwdcolor=#27a9ef
                     /ApEinfo_revcolor=#27a9ef
                     /ApEinfo_graphicformat=arrow_data {{0 1 2 0 0 -1} {}
0}
                     width 5 offset 0
     misc_feature    join(2151..2361,2363..2485)
                     /label=Fragment 1
                     /ApEinfo_fwdcolor=#cca633
                     /ApEinfo_revcolor=#cca633
                     /ApEinfo_graphicformat=arrow_data {{0 1 2 0 0 -1} {}
0}
                     width 5 offset 0
     CDS             complement(4736..5395)
                     /label=AmpR
                     /ApEinfo_fwdcolor=yellow
                     /ApEinfo_revcolor=yellow
                     /ApEinfo_graphicformat=arrow_data {{0 1 2 0 0 -1} {}
0}
                     width 5 offset 0
     rep_origin      complement(3956..4638)
```

```
                     /label=ColE1 origin
                     /ApEinfo_fwdcolor=gray50
                     /ApEinfo_revcolor=gray50
                     /ApEinfo_graphicformat=arrow_data {{0 1 2 0 0 -1} {}
0}
                     width 5 offset 0
     red             3603..3655
                     /label=Lambda t1 terminator
                     /ApEinfo_fwdcolor=red
                     /ApEinfo_revcolor=green
                     /ApEinfo_graphicformat=arrow_data {{0 1 2 0 0 -1} {}
0}
                     width 5 offset 0
     misc_feature    2991..3011
                     /label=lac O1
                     /ApEinfo_fwdcolor=#af6436
                     /ApEinfo_revcolor=#ff8040
                     /ApEinfo_graphicformat=arrow_data {{0 1 2 0 0 -1} {}
0}
                     width 5 offset 0
     misc_feature    2346..2352
                     /label=BstEII
                     /ApEinfo_fwdcolor=#ff80c0
                     /ApEinfo_revcolor=green
                     /ApEinfo_graphicformat=arrow_data {{0 1 2 0 0 -1} {}
0}
                     width 5 offset 0
     misc_feature    2351..2351
                     /label=stall site +22
                     /ApEinfo_fwdcolor=#f5010a
                     /ApEinfo_revcolor=green
                     /ApEinfo_graphicformat=arrow_data {{0 1 2 0 0 -1} {}
0}
                     width 5 offset 0
     misc_feature    2086..2105
                     /label=S/JBOIDO1_400/2086
                     /ApEinfo_fwdcolor=cyan
                     /ApEinfo_revcolor=green
                     /ApEinfo_graphicformat=arrow_data {{0 1 2 0 0 -1} {}
0}
                     width 5 offset 0
     misc_feature    complement(5096..5112)
                     /label=A/JBOIDO1_400/5096_Apa1
                     /ApEinfo_fwdcolor=cyan
                     /ApEinfo_revcolor=green
                     /ApEinfo_graphicformat=arrow_data {{0 1 2 0 0 -1} {}
0}
                     width 5 offset 0
     misc_feature    2330..2331
                     /label=start point
                     /ApEinfo_fwdcolor=#ff0000
                     /ApEinfo_revcolor=green
                     /ApEinfo_graphicformat=arrow_data {{0 1 2 0 0 -1} {}
0}
```

```
                     width 5 offset 0
     promoter        2277..2330
                     /label=T7A1
                     /ApEinfo_fwdcolor=#bbffbb
                     /ApEinfo_revcolor=#c1ffff
                     /ApEinfo_graphicformat=arrow_data {{0 1 2 0 0 -1} {}
0}
                     width 5 offset 0
     misc_feature    2592..2612
                     /label=O2
                     /ApEinfo_fwdcolor=#e3c5a2
                     /ApEinfo_revcolor=#dd7b22
                     /ApEinfo_graphicformat=arrow_data {{0 1 2 0 0 -1} {}
0}
                     width 5 offset 0
     misc_feature    2318..2323
                     /label=TATA Box
                     /ApEinfo_fwdcolor=#ff8000
                     /ApEinfo_revcolor=green
                     /ApEinfo_graphicformat=arrow_data {{0 1 2 0 0 -1} {}
0}
                     width 5 offset 0
ORIGIN
        1 tatcacagtt aaattgctaa cgcagtcagg caccgtgtat gaaatctaac
aatgcgctca
       61 tcgtcatcct cggcaccgtc accctggatg ctgtaggcat aggcttggtt
atgccggtac
      121 tgccgggcct cttgcgggat atcgtccatt ccgacagcat cgccagtcac
tatggcgtgc
      181 tgctagcgct atatgcgttg atgcaatttc tatgcgcacc cgttctcgga
gcactgtccg
      241 accgctttgg ccgccgccca gtcctgctcg cttcgctact tggagccact
atcgactacg
      301 cgatcatggc gaccacaccc gtcctgtgga tcctctacgc cggacgcatc
gtggccggca
      361 tcaccggcgc cacaggtgcg gttgctggcg cctatatcgc cgacatcacc
gatggggaag
      421 atcgggctcg ccacttcggg ctcatgagcg cttgtttcgg cgtgggtatg
gtggcaggcc
      481 ccgtggccgg gggactgttg ggcgccatct ccttgcatgc accattcctt
gcggcggcgg
      541 tgctcaacgg cctcaaccta ctactgggct gcttcctaat gcaggagtcg
cataagggag
      601 agcgtcgacc gatgcccttg agagccttca acccagtcag ctccttccgg
tgggcgcggg
      661 gcatgactat cgtcgccgca cttatgactg tcttctttat catgcaactc
gtaggacagg
      721 tgccggcagc gctctgggtc attttcggcg aggaccgctt tcgctggagc
gcgacgatga
      781 tcggcctgtc gcttgcggta ttcggaatct tgcacgccct cgctcaagcc
ttcgtcactg
      841 gtcccgccac caaacgtttc ggcgagaagc aggccattat cgccggcatg
gcggccgacg
```

901 cgctgggcta cgtcttgctg gcgttcgcga cgcgaggctg gatggccttc cccattatga
961 ttcttctcgc ttccggcggc atcgggatgc ccgcgttgca ggccatgctg tccaggcagg
1021 tagatgacga ccatcaggga cagcttcaag gatcgctcgc ggctcttacc agcctaactt
1081 cgatcattgg accgctgatc gtcacggcga tttatgccgc ctcggcgagc acatggaacg
1141 ggttggcatg gattgtaggc gccgccctat accttgtctg cctccccgcg ttgcgtcgcg
1201 gtgcatggag ccgggccacc tcgacctgaa tggaagccgg cggcacctcg ctaacggatt
1261 caccactcca agaattggag ccaatcaatt cttgcggaga actgtgaatg cgcaaaccaa
1321 cccttggcag aacatatcca tcgcgtccgc catctccagc agccgcacgc ggcgcatctc
1381 gggcagcgtt gggtcctggc cacgggtgcg catgatcgtg ctcctgtcgt tgaggacccg
1441 gctaggctgg cggggttgcc ttactggtta gcagaatgaa tcaccgatac gcgagcgaac
1501 gtgaagcgac tgctgctgca aaacgtctgc gacctgagca acaacatgaa tggtcttcgg
1561 tttccgtgtt tcgtaaagtc tggaaacgcg gaagtcagcg ccctgcacca ttatgttccg
1621 gatctgcatc gcaggatgct gctggctacc ctgtggaaca cctacatctg tattaacgaa
1681 gcgctggcat tgaccctgag tgatttttct ctggtcccgc cgcatccata ccgccagttg
1741 tttaccctca caacgttcca gtaaccgggc atgttcatca tcagtaaccc gtatcgtgag
1801 catcctctct cgtttcatcg gtatcattac ccccatgaac agaaatcccc cttacacgga
1861 ggcatcagtg accaaacagg aaaaaaccgc ccttaacatg gcccgcttta tcagaagcca
1921 gacattaacg cttctggaga aactcaacga gctggacgcg gatgaacagg cagacatctg
1981 tgaatcgctt cacgaccacg ctgatgagct ttaccgcagc tgcctcgcgc gtttcggtga
2041 tgacggtgaa aacctctgac acatgcagct cccggagacg gtcacagctt gtctgtaagc
2101 ggatgccggg agcagacaag cccgtcaggg cgcgtcagcg ggtgttggcA ggtgtcgggg
2161 cgcagccatg acccagtcac cccatggtgc agtatgaagg cggcggagcc gacaccacgg
2221 ccaccgatat tatttgcccg atgtacgcgc gcgtggatga agaccagccc ttcccggctt
2281 tatcaaaaag agtattgact taaagtctaa cctataggat acttacagcg atggagaggt
2341 gtagtggtaa ccagaagata aTatggcttt cgctacctgg agagacgcgc ccgctgatcc
2401 tttgcgaata cgcccacgcg atgggtaaca gtcttggcgg tttcgctaaa tactggcagg
2461 cgtttcgtca gtatccccgt ttacagggcg gcttcgtctg ggactgggtg gatcagtcgc

2521 tgattaaata tgatgaaaac ggcaacccgt ggtaccggct tacggcggtg atttTgcga
2581 tacgccgaac gaaatgtgag cgagtaacaa ccatcgccag ttctgtatga acggtctggt
2641 ctttgccgac cgcacgccgc atccagcgct gacggaagca aaacaccagc agcagttttt
2701 cagttccgtt tatccgggca aaccatcgaa gtgaccagcg aatacctgtt ccgtcatagc
2761 gataacgagc tcctgcactg gatggtggcg ctggatggta agccgctggc aagcggtgaa
2821 gtgcctctgg atgtcgctcc acaaggtaaa cagttgattg aactgTctga actaccgcag
2881 ccggagagcg ccgggcaact ctggctcaca gtacgcgtag tgcaaccgaa cgcgaccgca
2941 tggtcagaag ccgggcacat cagcgcctgg cagcagtAgc gtctggcgga aattgtgagc
3001 ggataacaat taaacctcag tgtgacgctc cccgccgcgt cccacgccat cccgcatctg
3061 accaccagcg aaatggattt ttgcactcga gctgggtaat aagcgttggc aatttaaccg
3121 ccagtcaggc tttctttcac agatgtggat tggcgataaa aaacaactgc tgacgccgct
3181 gcgcgatcag ttcacccgtg caccgctgga taacgacatt ggcgtaagtg aagcgacccg
3241 cattgaccct aacgcctggg tcgaacActg gaaggcggcg ggccattacc aggccgaagc
3301 agcgttgttg cagtgcacgg cagatacact tgctgatgcg gtgctgatta cgaccgctca
3361 cgcgtggcag catcagggga aaaccttatt tatcagccgg aaaacctacc ggattgatgg
3421 tagtggtcaa atggcgatta ccgttgatgt tgaagtggcg agcgatacac cgcatccggc
3481 gcggattggc ctgaactgcc agctggcgca ggtagcagag cgggtaaact ggctcggatt
3541 agcggccgca agaaaactat cccgaccgcc ttactgccgc ctgttttgac cgctgggatc
3601 tgctgtaaca gagcattagc gcaaggtgat tttgtcttc ttgcgctaat tttttccatt
3661 gtctagagta gcgatagcgg agtgtatact ggcttaacta tgcggcatca gagcagattg
3721 tactgagagt gcaccatatg cggtgtgaaa taccgcacag atgcgtaagg agaaaatacc
3781 gcatcaggcg ctcttccgct tcctcgctca ctgactcgct gcgctcggtc gttcggctgc
3841 ggcgagcggt atcagctcac tcaaaggcgg taatacggtt atccacagaa tcaggggata
3901 acgcaggaaa gaacatgtga gcaaaaggcc agcaaaaggc caggaaccgt aaaaaggccg
3961 cgttgctggc gtttttccat aggctccgcc cccctgacga gcatcacaaa aatcgacgct
4021 caagtcagag gtggcgaaac ccgacaggac tataaagata ccaggcgttt ccccctggaa
4081 gctccctcgt gcgctctcct gttccgaccc tgccgcttac cggatacctg tccgcctttc

4141 tcccttcggg aagcgtggcg ctttctcata gctcacgctg taggtatctc agttcggtgt
4201 aggtcgttcg ctccaagctg ggctgtgtgc acgaaccccc cgttcagccc gaccgctgcg
4261 ccttatccgg taactatcgt cttgagtcca acccggtaag acacgactta tcgccactgg
4321 cagcagccac tggtaacagg attagcagag cgaggtatgt aggcggtgct acagagttct
4381 tgaagtggtg gcctaactac ggctacacta gaaggacagt atttggtatc tgcgctctgc
4441 tgaagccagt taccttcgga aaaagagttg gtagctcttg atccggcaaa caaaccaccg
4501 ctggtagcgg tggttttttt gtttgcaagc agcagattac gcgcagaaaa aaaggatctc
4561 aagaagatcc tttgatcttt tctacggggt ctgacgctca gtggaacgaa aactcacgtt
4621 aagggatttt ggtcatgaga ttatcaaaaa ggatcttcac ctagatcctt ttaaattaaa
4681 aatgaagttt taaatcaatc taaagtatat atgagtaaac ttggtctgac agttaccaat
4741 gcttaatcag tgaggcacct atctcagcga tctgtctatt tcgttcatcc atagttgcct
4801 gactccccgt cgtgtagata actacgatac gggagggctt accatctggc cccagtgctg
4861 caatgatacc gcgagaccca cgctcaccgg ctccagattt atcagcaata aaccagccag
4921 ccggaagggc cgagcgcaga agtggtcctg caactttatc cgcctccatc cagtctatta
4981 attgttgccg ggaagctaga gtaagtagtt cgccagttaa tagtttgcgc aacgttgttg
5041 ccattgctgc aggcatcgtg gtgtcacgct cgtcgtttgg tatggcttca ttcagctccg
5101 gttcccaacg atcaaggcga gttacatgat cccccatgtt gtgcaaaaaa gcggttagct
5161 ccttcggtcc tccgatcgtt gtcagaagta agttggccgc agtgttatca ctcatggtta
5221 tggcagcact gcataattct cttactgtca tgccatccgt aagatgcttt tctgtgactg
5281 gtgagtactc aaccaagtca ttctgagaat agtgtatgcg gcgaccgagt tgctcttgcc
5341 cggcgtcaac acgggataat accgcgccac atagcagaac tttaaaagtg ctcatcattg
5401 gaaaacgttc ttcggggcga aaactctcaa ggatcttacc gctgttgaga tccagttcga
5461 tgtaacccac tcgtgcaccc aactgatctt cagcatcttt tactttcacc agcgtttctg
5521 ggtgagcaaa aacaggaagg caaaatgccg caaaaaaggg aataagggcg acacggaaat
5581 gttgaatact catactcttc ctttttcaat attattgaag catttatcag ggttattgtc
5641 tcatgagcgg atacatattt gaatgtattt agaaaaataa acaaataggg gttccgcgca
5701 catttccccg aaaagtgcca cctgacgtct aagaaaccat tattatcatg acattaacct

```
     5761 ataaaaatag gcgtatcacg aggccctttc gtcttcaaga attctcatgt
ttgacagctt
     5821 atcatcgata agctttaatg cggtagtt
//
```
